# A Learning Theory for Reward-Modulated Spike-Timing-Dependent Plasticity with Application to Biofeedback

**DOI:** 10.1371/journal.pcbi.1000180

**Published:** 2008-10-10

**Authors:** Robert Legenstein, Dejan Pecevski, Wolfgang Maass

**Affiliations:** Institute for Theoretical Computer Science, Graz University of Technology, Graz, Austria; UFR Biomédicale de l'Université René Descartes, France

## Abstract

Reward-modulated spike-timing-dependent plasticity (STDP) has recently emerged as a candidate for a learning rule that could explain how behaviorally relevant adaptive changes in complex networks of spiking neurons could be achieved in a self-organizing manner through local synaptic plasticity. However, the capabilities and limitations of this learning rule could so far only be tested through computer simulations. This article provides tools for an analytic treatment of reward-modulated STDP, which allows us to predict under which conditions reward-modulated STDP will achieve a desired learning effect. These analytical results imply that neurons can learn through reward-modulated STDP to classify not only spatial but also temporal firing patterns of presynaptic neurons. They also can learn to respond to specific presynaptic firing patterns with particular spike patterns. Finally, the resulting learning theory predicts that even difficult credit-assignment problems, where it is very hard to tell which synaptic weights should be modified in order to increase the global reward for the system, can be solved in a self-organizing manner through reward-modulated STDP. This yields an explanation for a fundamental experimental result on biofeedback in monkeys by Fetz and Baker. In this experiment monkeys were rewarded for increasing the firing rate of a particular neuron in the cortex and were able to solve this extremely difficult credit assignment problem. Our model for this experiment relies on a combination of reward-modulated STDP with variable spontaneous firing activity. Hence it also provides a possible functional explanation for trial-to-trial variability, which is characteristic for cortical networks of neurons but has no analogue in currently existing artificial computing systems. In addition our model demonstrates that reward-modulated STDP can be applied to all synapses in a large recurrent neural network without endangering the stability of the network dynamics.

## Author Summary

A major open problem in computational neuroscience is to explain how learning, i.e., behaviorally relevant modifications in the central nervous system, can be explained on the basis of experimental data on synaptic plasticity. Spike-timing-dependent plasticity (STDP) is a rule for changes in the strength of an individual synapse that is supported by experimental data from a variety of species. However, it is not clear how this synaptic plasticity rule can produce meaningful modifications in networks of neurons. Only if one takes into account that consolidation of synaptic plasticity requires a third signal, such as changes in the concentration of a neuromodulator (that might, for example, be related to rewards or expected rewards), then meaningful changes in the structure of networks of neurons may occur. We provide in this article an analytical foundation for such reward-modulated versions of STDP that predicts when this type of synaptic plasticity can produce functionally relevant changes in networks of neurons. In particular we show that seemingly inexplicable experimental data on biofeedback, where a monkey learnt to increase the firing rate of an arbitrarily chosen neuron in the motor cortex, can be explained on the basis of this new learning theory.

## Introduction

Numerous experimental studies (see [1] for a review; [2] discusses more recent in-vivo results) have shown that the efficacy of synapses changes in dependence of the time difference Δ*t* = *t_post_*−*t_pre_* between the firing times *t_pre_* and *t_post_* of the pre- and postsynaptic neurons. This effect is called spike-timing-dependent plasticity (STDP). But a major puzzle for understanding learning in biological organisms is the relationship between experimentally well-established rules for STDP on the microscopic level, and adaptive changes of the behavior of biological organisms on the macroscopic level. Neuromodulatory systems, which send diffuse signals related to reinforcements (rewards) and behavioral state to several large networks of neurons in the brain, have been identified as likely intermediaries that relate these two levels of plasticity. It is well-known that the consolidation of changes of synaptic weights in response to pre- and postsynaptic neuronal activity requires the presence of such third signals [3],[4]. In particular, it has been demonstrated that dopamine (which is behaviorally related to novelty and reward prediction [5]) gates plasticity at corticostriatal synapses [6],[7] and within the cortex [8]. It has also been shown that acetylcholine gates synaptic plasticity in the cortex (see for example [9] and [10],[11] contains a nice review of the literature).

**Figure 1:**
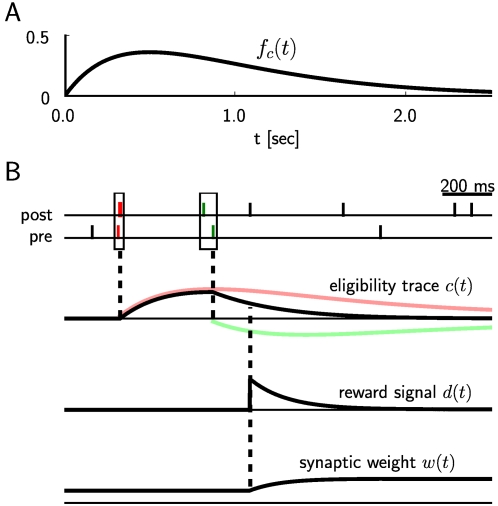
Scheme of reward-modulated STDP according to Equations 1–4. (A) Eligibility function *f_c_*(*t*), which scales the contribution of a pre/post spike pair (with the second spike at time 0) to the eligibility trace *c*(*t*) at time *t*. (B) Contribution of a pre-before-post spike pair (in red) and a post-before-pre spike pair (in green) to the eligibility trace *c*(*t*) (in black), which is the sum of the red and green curves. According to Equation 1 the change of the synaptic weight *w* is proportional to the product of *c*(*t*) with a reward signal *d*(*t*).

Corresponding spike-based rules for synaptic plasticity of the form
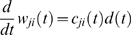
(1)have been proposed in [12] and [13] (see Figure 1 for an illustration of this learning rule), where *w_ji_* is the weight of a synapse from neuron *i* to neuron *j*, *c_ji_*(*t*) is an eligibility trace of this synapse which collects weight changes proposed by STDP, and *d*(*t*) = *h*(*t*)−*h̅* results from a neuromodulatory signal *h*(*t*) with mean value *h̅*. It was shown in [12] that a number of interesting learning tasks in large networks of neurons can be accomplished with this simple rule in Equation 1. It has recently been shown that quite similar learning rules for spiking neurons arise when one applies the general framework of distributed reinforcement learning from [14] to networks of spiking neurons [13],[15], or if one maximizes the likelihood of postsynaptic firing at desired firing times [16]. However no analytical tools have been available, which make it possible to predict for what learning tasks, and under which parameter settings, reward-modulated STDP will be successful. This article provides such analytical tools, and demonstrates their applicability and significance through a variety of computer simulations. In particular, we identify conditions under which neurons can learn through reward-modulated STDP to classify temporal presynaptic firing patterns, and to respond with particular spike patterns.

We also provide a model for the remarkable operant conditioning experiments of [17] (see also [18],[19]). In the simpler ones of these experiments the spiking activity of single neurons (in area 4 of the precentral gyrus of monkey cortex) was recorded, the deviation of the current firing rate of an arbitrarily selected neuron from its average firing rate was made visible to the monkey through the displacement of an illuminated meter arm, whose rightward position corresponded to the threshold for the feeder discharge. The monkey received food rewards for increasing (or in alternating trials for decreasing) the firing rate of this neuron. The monkeys learnt quite reliably (within a few minutes) to change the firing rate of this neuron in the currently rewarded direction. Adjacent neurons tended to change their firing rate in the same direction, but also differential changes of directions of firing rates of pairs of neurons are reported in [17] (when these differential changes were rewarded). For example, it was shown in Figure 9 of [17] (see also Figure 1 in [19]) that pairs of neurons that were separated by no more than a few hundred microns could be independently trained to increase or decrease their firing rates. Obviously the existence of learning mechanisms in the brain which are able to solve this extremely difficult credit assignment problem provides an important clue for understanding the organization of learning in the brain. We examine in this article analytically under what conditions reward-modulated STDP is able to solve such learning problem. We test the correctness of analytically derived predictions through computer simulations of biologically quite realistic recurrently connected networks of neurons, where an increase of the firing rate of one arbitrarily selected neuron within a network of 4000 neurons is reinforced through rewards (which are sent to all 142813 synapses between excitatory neurons in this recurrent network). We also provide a model for the more complex operant conditioning experiments of [17] by showing that pairs of neurons can be differentially trained through reward-modulated STDP, where one neuron is rewarded for increasing its firing rate, and simultaneously another neuron is rewarded for decreasing its firing rate. More precisely, we increased the reward signal *d*(*t*) which is transmitted to all synapses between excitatory neurons in the network whenever the first neuron fired, and decreased this reward signal whenever the second neuron fired (the resulting composed reward corresponds to the displacement of the meter arm that was shown to the monkey in these more complex operant conditioning experiments).

Our theory and computer simulations also show that reward-modulated STDP can be applied to all synapses within a large network of neurons for long time periods, without endangering the stability of the network. In particular this synaptic plasticity rule keeps the network within the asynchronous irregular firing regime, which had been described in [20] as a dynamic regime that resembles spontaneous activity in the cortex. Another interesting aspect of learning with reward-modulated STDP is that it requires spontaneous firing and trial-to-trial variability within the networks of neurons where learning takes place. Hence our learning theory for this synaptic plasticity rule provides a foundation for a functional explanation of these characteristic features of cortical network of neurons that are undesirable from the perspective of most computational theories.

## Results

We first give a precise definition of the learning rule in Equation 1 for reward-modulated STDP. The standard rule for STDP, which specifies the change *W*(Δ*t*) of the synaptic weight of an excitatory synapse in dependence on the time difference Δ*t* = *t_post_*−*t_pre_* between the firing times *t_pre_* and *t_post_* of the pre- and postsynaptic neuron, is based on numerous experimental data (see [1]). It is commonly modeled by a so-called learning curve of the form
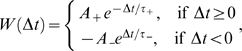
(2)where the positive constants *A*_+_ and *A*_−_ scale the strength of potentiation and depression respectively, and *τ*_+_ and *τ*_−_ are positive time constants defining the width of the positive and negative learning window. The resulting weight change at time *t* of synapse *ji* for a presynaptic spike train 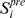 and a postsynaptic spike train 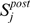 is usually modeled [21] by the instantaneous application of this learning rule to all spike pairings with the second spike at time *t*

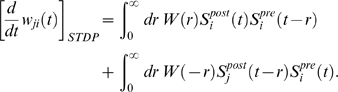
(3)The spike train of a neuron *i* which fires action potentials at times 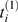, 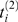, 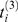,… is formalized here by a sum of Dirac delta functions 
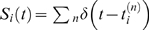
.

The model analyzed in this article is based on the assumption that positive and negative weight changes suggested by STDP for all pairs of pre- and postsynaptic spikes at synapse *ji* (according to the two integrals in Equation 3) are collected in an eligibility trace *c_ji_*(*t*) at the site of the synapse. The contribution to *c_ij_*(*t*) of all spike pairings with the second spike at time *t*−*s* is modeled for *s*>0 by a function *f_c_*(*s*) (see Figure 1A); the time scale of the eligibility trace is assumed in this article to be on the order of seconds. Hence the value of the eligibility trace of synapse *ji* at time *t* is given by
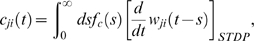
(4)see Figure 1B. The actual weight change 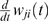 at time *t* for reward-modulated STDP is the product *c_ij_*(*t*)·*d*(*t*) of the eligibility trace with the reward signal *d*(*t*) as defined by Equation 1. Since this simple model can in principle lead to unbounded growth of weights, we assume that weights are clipped at the lower boundary value 0 and an upper boundary *w_max_*.

The network dynamics of a simulated recurrent network of spiking neurons where all connections between excitatory neurons are subject to STDP is quite sensitive to the particular STDP-rule that is used. Therefore we have carried out our network simulations not only with the additive STDP-rule in Equation 3, whose effect can be analyzed theoretically, but also with the more complex rule proposed in [22] (which was fitted to experimental data from hippocampal neurons in culture [23]), where the magnitude of the weight change depends on the current value of the weight. An implementation of this STDP-rule (with the parameters proposed in [22]) produced in our network simulations of the biofeedback experiment (computer simulation 1) as well as for learning pattern classification (computer simulation 4) qualitatively the same result as the rule in Equation 3.

### Theoretical Analysis of the Resulting Weight Changes

In this section, we derive a learning equation for reward-modulated STDP. This learning equation relates the change of a synaptic weight *w_ji_* over some sufficiently long time interval *T* to statistical properties of the joint distribution of the reward signal *d*(*t*) and pre- and postsynaptic firing times, under the assumption that the weight and correlations between pre- and postsynaptic spike times are slowly varying in time. We treat spike times as well as the reward signal *d*(*t*) as stochastic variables. This mathematical framework allows us to derive the expected weight change over some time interval *T* (see [21]), with the expectation taken over realizations of the stochastic input- and output spike trains as well as stochastic realizations of the reward signal, denoted by the ensemble average 〈·〉*_E_*

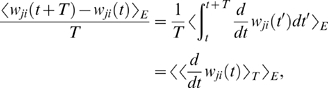
(5)where we used the abbreviation 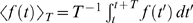. If synaptic plasticity is sufficiently slow, synaptic weights integrate a large number of small changes. In this case, the weight *w_ji_* can be approximated by its average 〈*w_ji_*〉*_E_* (it is “self-averaging”, see [21]). We can thus drop the expectation on the left hand side of Equation 5 and write it as 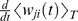. Using Equation 1, this yields (see Methods)
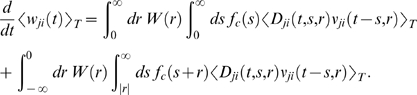
(6)This formula contains the *reward correlation* for synapse *ji*

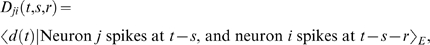
(7)which is the average reward at time *t* given a presynaptic spike at time *t*−*s*−*r* and a postsynaptic spike at time *t*−*s*. The joint firing rate *ν_ji_*(*t*,*r*) = 〈*S_j_*(*t*)*S_i_*(*t*−*r*)〉*_E_* describes correlations between spike timings of neurons *j* and *i*, i.e., it is the probability density for the event that neuron *i* fires an action potential at time *t*−*r* and neuron *j* fires an action potential at time *t*. For synapses subject to reward-modulated STDP, changes in efficacy are obviously driven by co-occurrences of spike pairings and rewards within the time scale of the eligibility trace. Equation 6 clarifies how the expected weight change depends on how the correlations between the pre- and postsynaptic neurons correlate with the reward signal.

If one assumes for simplicity that the impact of a spike pair on the eligibility trace is always triggered by the postsynaptic spike, one gets a simpler equation (see Methods)
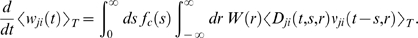
(8)The assumption introduces a small error for post-before-pre spike pairs, because for a reward signal that arrives at some time *d_r_* after the pairing, the weight update will be proportional to *f_c_*(*d_r_*) instead of *f_c_*(*d_r_*+*r*). The approximation is justified if the temporal average is performed on a much longer time scale than the time scale of the learning window, the effect of each pre-post spike pair on the reward signal is delayed by an amount greater than the time scale of the learning window, and *f_c_* changes slowly compared to the time scale of the learning window (see Methods for details). For the analyzes presented in this article, the simplified Equation 8 is a good approximation for the learning dynamics. Equation 8 is a generalized version of the STDP learning equation 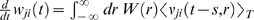 in [21] that includes the impact of the reward correlation weighted by the eligibility function. To see the relation between standard STDP and reward-modulated STDP, consider a constant reward signal *d*(*t*) = *d*_0_. Then also the reward correlation is constant and given by *D*(*t*,*s*,*r*) = *d*_0_. We recover the standard STDP learning equation scaled by *d*_0_ if the eligibility function is an instantaneous delta-pulse *f_c_*(*s*) = *δ*(*s*). Furthermore, if the statistics of the reward signal *d*(*t*) is time-independent and independent from the pre- and postsynaptic spike statistics of some synapse *ji*, then the reward correlation is given by *D_ji_*(*t*,*s*,*r*) = 〈*d*(*t*)〉*_E_* = *d*_0_ for some constant *d*_0_. Then, the weight change for synapse *ji* is 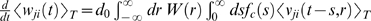. The temporal average of the joint firing rate 〈*ν_ji_*(*t*−*s*,*r*〉*_T_* is thus filtered by the eligibility trace. We assumed in the preceding analysis that the temporal average is taken over some long time interval *T*. If the time scale of the eligibility trace is much smaller than this time interval *T*, then the weight change is approximately 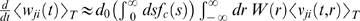, and the weight *w_ji_* will change according to standard STDP scaled by a constant proportional to the mean reward and the integral over the eligibility function. In the remainder of this article, we will always use the smooth time-averaged weight change 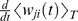, but for brevity, we will drop the angular brackets and simply write 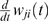.

The learning Equation 8 provides the mathematical basis for our following analyses. It allows us to determine synaptic weight changes if we can describe a learning situation in terms of reward correlations and correlations between pre- and postsynaptic spikes.

### Application to Models for Biofeedback Experiments

We now apply the preceding analysis to the biofeedback experiment of [17] that were described in the introduction. These experiments pose the challenge to explain how learning mechanisms in the brain can detect and exploit correlations between rewards and the firing activity of one or a few neurons within a large recurrent network of neurons (the credit assignment problem), without changing the overall function or dynamics of the circuit.

**Figure 2:**
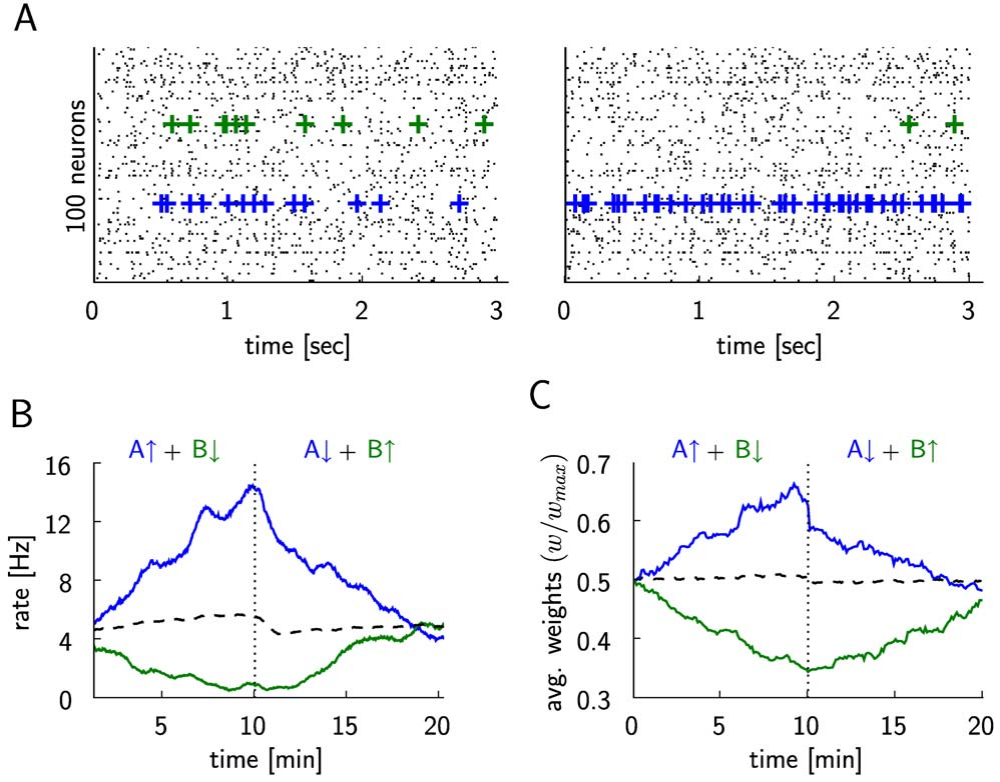
Differential reinforcement of two neurons (within a simulated network of 4000 neurons, the two rewarded neurons are denoted as A and B), corresponding to the experimental results shown in Figure 9 of [17] and Figure 1 of [19]. (A) The spike response of 100 randomly chosen neurons at the beginning of the simulation (20 sec–23 sec, left plot), and at the middle of simulation just before the switching of the reward policy (597 sec–600 sec, right plot). The firing times of the first reinforced neuron A are marked by blue crosses and those of the second reinforced neuron B are marked by green crosses. (B) The dashed vertical line marks the switch of the reinforcements at *t* = 10 min. The firing rate of neuron A (blue line) increases while it is positively reinforced in the first half of the simulation and decreases in the second half when its spiking is negatively reinforced. The firing rate of the neuron B (green line) decreases during the negative reinforcement in the first half and increases during the positive reinforcement in the second half of the simulation. The average firing rate of 20 other randomly chosen neurons (dashed line) remains unchanged. (C) Evolution of the average weight of excitatory synapses to the rewarded neurons A and B (blue and green lines, respectively), and of the average weight of 1744 randomly chosen excitatory synapses to other neurons in the circuit (dashed line).

**Figure 3:**
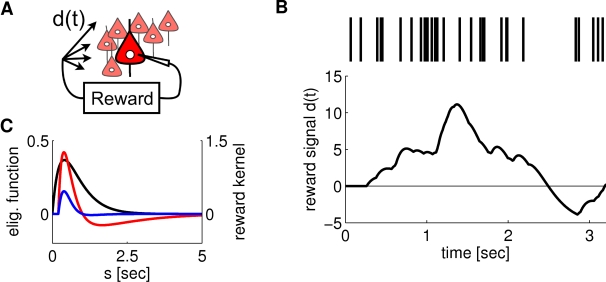
Setup of the model for the experiment by Fetz and Baker [17]. (A) Schema of the model: The activity of a single neuron in the circuit determines the amount of reward delivered to all synapses between excitatory neurons in the circuit. (B) The reward signal *d*(*t*) in response to a spike train (shown at the top) of the arbitrarily selected neuron (which was selected from a recurrently connected circuit consisting of 4000 neurons). The level of the reward signal *d*(*t*) follows the firing rate of the spike train. (C) The eligibility function *f_c_*(*s*) (black curve, left axis), the reward kernel *ε_r_*(*s*) delayed by 200 ms (red curve, right axis), and the product of these two functions (blue curve, right axis) as used in our computer experiment. The integral of *f_c_*(*s*+*d_r_*)*ε_r_*(*s*) is positive, as required according to Equation 10 in order to achieve a positive learning rate for the synapses to the selected neuron.

We show that this phenomenon can in principle be explained by reward-modulated STDP. In order to do that, we define a model for the experiment which allows us to formulate an equation for the reward signal *d*(*t*). This enables us to calculate synaptic weight changes for this particular scenario. We consider as model a recurrent neural circuit where the spiking activity of one neuron *k* is recorded by the experimenter (Experiments where two neurons are recorded and reinforced were also reported in [17]. We tested this case in computer simulations (see Figure 2) but did not treat it explicitly in our theoretical analysis). We assume that in the monkey brain a reward signal *d*(*t*) is produced which depends on the visual feedback (through an illuminated meter, whose pointer deflection was dependent on the current firing rate of the randomly selected neuron *k*) as well as previously received liquid rewards, and that this signal *d*(*t*) is delivered to *all* synapses in large areas of the brain. We can formalize this scenario by defining a reward signal which depends on the spike rate of the arbitrarily selected neuron *k* (see Figure 3A and 3B). More precisely, a reward pulse of shape *ε_r_*(*r*) (the reward kernel) is produced with some delay *d_r_* every time the neuron *k* produces an action potential
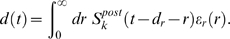
(9)Note that *d*(*t*) = *h*(*t*)−*h̅* is defined in Equation 1 as a signal with zero mean. In order to satisfy this constraint, we assume that the reward kernel *ε_r_* has zero mass, i.e., 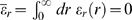. For the analysis, we use the linear Poisson neuron model described in Methods. The mean weight change for synapses to the reinforced neuron *k* is then approximately (see Methods)
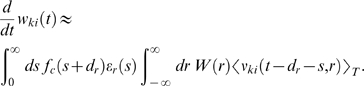
(10)This equation describes STDP with a learning rate proportional to 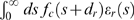. The outcome of the learning session will strongly depend on this integral and thus on the form of the reward kernel *ε_r_*. In order to reinforce high firing rates of the reinforced neuron we have chosen a reward kernel with a positive bump in the first few hundred milliseconds, and a long negative tail afterwards. Figure 3C shows the functions *f_c_* and *ε_r_* that were used in our computer model, as well as the product of these two functions. One sees that the integral over the product is positive and according to Equation 10 the synapses to the reinforced neuron are subject to STDP. This does not guarantee an increase of the firing rate of the reinforced neuron. Instead, the changes of neuronal firing will depend on the statistics of the inputs. In particular, the weights of synapses to neuron *k* will not increase if that neuron does not fire spontaneously. For uncorrelated Poisson input spike trains of equal rate, the firing rate of a neuron trained by STDP stabilizes at some value which depends on the input rate (see [24],[25]). However, in comparison to the low spontaneous firing rates observed in the biofeedback experiment [17], the stable firing rate under STDP can be much higher, allowing for a significant rate increase. It was shown in [17] that also low firing rates of a single neuron can be reinforced. In order to model this, we have chosen a reward kernel with a negative bump in the first few hundred milliseconds, and a long positive tail afterwards, i.e. we inverted the kernel used above to obtain a negative integral 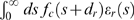. According to Equation 10 this leads to anti-STDP where not only inputs to the reinforced neuron which have low correlations with the output are depressed (because of the negative integral of the learning window), but also those which are causally correlated with the output. This leads to a quick firing rate decrease at the reinforced neuron.

The mean weight change of synapses to non-reinforced neurons *j*≠*k* is given by
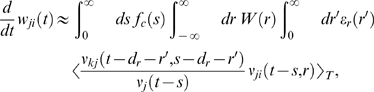
(11)where *ν_j_*(*t*) = 〈*S_j_*(*t*)〉*_E_* is the instantaneous firing rate of neuron *j* at time *t*. This equation indicates that a non-reinforced neuron is trained by STDP with a learning rate proportional to its correlation with the reinforced neuron given by *ν_kj_*(*t*−*d_r_*−*r*′,*s*−*d_r_*−*r*′)/*ν_j_*(*t*−*s*). In fact, it was noted in [17] that neurons nearby the reinforced neuron tended to change their firing rate in the same direction. This observation might be explained by putative correlations of the recorded neuron with nearby neurons. On the other hand, if a neuron *j* is uncorrelated with the reinforced neuron *k*, we can decompose the joint firing rate into *ν_kj_*(*t*−*d_r_*−*r*′,*s*−*d_r_*−*r*′) = *ν_k_*(*t*−*d_r_*−*r*′)*ν_j_*(*t*−*s*). In this case, the learning rate for synapse *ji* is approximately zero (see Methods). This ensures that most neurons in the circuit keep a constant firing rate, in spite of continuous weight changes according to reward-modulated STDP.

Altogether we see that the weights of synapses to the reinforced neuron *k* can only change if there is spontaneous activity in the network, so that in particular also this neuron *k* fires spontaneously. On the other hand the spontaneous network activity should not consist of repeating large-scale spatio-temporal firing patterns, since that would entail correlations between the firing of neuron *k* and other neurons *j*, and would lead to similar changes of synapses to these other neurons *j*. Apart from these requirements on the spontaneous network activity, the preceding theoretical results predict that stability of the circuit is preserved, while the neuron which is causally related to the reward signal is trained by STDP, if 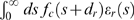 is positive.

### Computer Simulation 1: Model for Biofeedback Experiment

We tested these theoretical predictions through computer simulations of a generic cortical microcircuit receiving a reward signal which depends on the firing of one arbitrarily chosen neuron *k* from the circuit (reinforced neuron). The circuit was composed of 4000 LIF neurons, with 3200 being excitatory and 800 inhibitory, interconnected randomly by 228954 conductance based synapses with short term dynamics (All computer simulations were also carried out as a control with static current based synapses, see Methods and Suppl.). In addition to the explicitly modeled synaptic connections, conductance noise (generated by an Ornstein-Uhlenbeck process) was injected into each neuron according to data from [26], in order to model synaptic background activity of neocortical neurons in-vivo (More precisely, for 50% of the excitatory neurons the amplitude of the noise injection was reduced to 20%, and instead their connection probabilities from other excitatory neurons were chosen to be larger, see Methods and Figure S1 and Figure S2 for details. The reinforced neuron had to be chosen from the latter population, since reward-modulated STDP does not work properly if the postsynaptic neuron fires too often because of directly injected noise). This background noise elicited spontaneous firing in the circuit at about 4.6 Hz. Reward-modulated STDP was applied continuously to all synapses which had excitatory presynaptic and postsynaptic neurons, and all these synapses received the same reward signal. The reward signal was modeled according to Equation 9. Figure 3C shows one reward pulse caused by a single postsynaptic spike at time *t* = 0 with the parameters used in the experiment. For several postsynaptic spikes, the amplitude of the reward signal follows the firing rate of the reinforced neuron, see Figure 3B.

**Figure 4:**
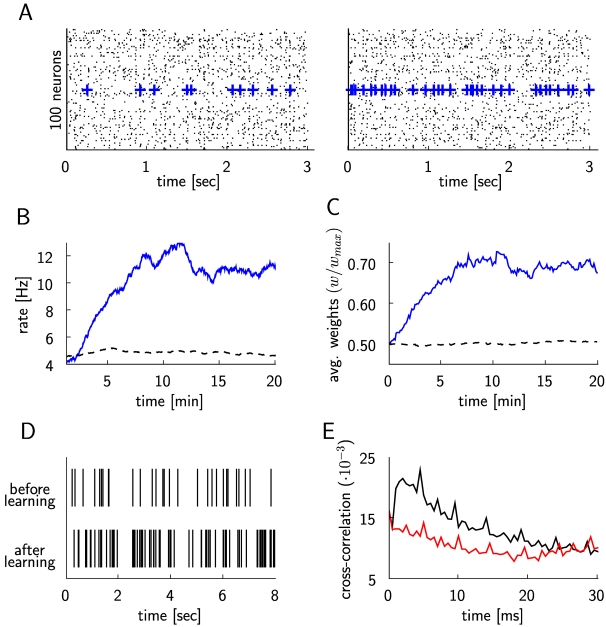
Simulation of the experiment by Fetz and Baker [17] for the case where an arbitrarily selected neuron triggers global rewards when it increases its firing rate. (A) Spike response of 100 randomly chosen neurons within the recurrent network of 4000 neurons at the beginning of the simulation (20 sec–23 sec, left plot), and at the end of the simulation (the last 3 seconds, right plot). The firing times of the reinforced neuron are marked by blue crosses. (B) The firing rate of the positively rewarded neuron (blue line) increases, while the average firing rate of 20 other randomly chosen neurons (dashed line) remains unchanged. (C) Evolution of the average weight of excitatory synapses to the reinforced neuron (blue line), and of the average weight of 1663 randomly chosen excitatory synapses to other neurons in the circuit (dashed line). (D) Spike trains of the reinforced neuron before and after learning. (E) Histogram of the time-differences between presynaptic and postsynaptic spikes (bin size 0.5 ms), averaged over all excitatory synapses to the reinforced neuron. The black curve represents the histogram values for positive time differences (when the presynaptic spike precedes the postsynaptic spike), and the red curve represents the histogram for negative time differences.

This model was simulated for 20 minutes of biological time. Figure 4A, 4B, and 4D show that the firing rate of the reinforced neuron increases within a few minutes (like in the experiment of [17]), while the firing rates of the other neurons remain largely unchanged. The increase of weights to the reinforced neuron shown in Figure 4C can be explained by the correlations between its presynaptic and postsynaptic spikes shown in panel E. This panel shows that pre-before-post spike pairings (black curve) are in general more frequent than post-before-pre spike pairings. The reinforced neuron increases its rate from around 4 Hz to 12 Hz, which is comparable to the measured firing rates in [15] before and after learning.

In Figure 9 of [17] and Figure 1 of [19] the results of another experiment were reported where the activity of two adjacent neurons was recorded, and high firing rates of the first neuron and low firing rates of the second neuron were reinforced simultaneously. This kind of differential reinforcement resulted in an increase and decrease of the firing rates of the two neurons correspondingly. We implemented this type of reinforcement by letting the reward signal in our model depend on the spikes of the two randomly chosen neurons (we refer to these neurons as neuron A and neuron B), i.e. 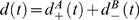, where 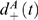 is the component that positively rewards spikes of neuron A, and 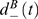 negatively rewards spikes of neuron B. Both parts of the reward signal, 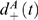 and 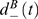, were defined as in Equation 9 for the corresponding neuron. For 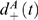 we used the reward kernel *ε_r_* as defined in Equation 29, whereas for 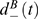 we used *ε_r_*_−_ = −*ε_r_* (note that the integral over *ε_r_*_−_ is still zero). At the middle of the simulation (simulation time *t* = 10 min), we changed the direction of the reinforcements by negatively rewarding the firing of neuron A and positively rewarding the firing of neuron B (i.e., 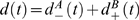). The results are summarized in Figure 2. With a reward signal modeled in this way, we were able to independently increase and decrease the firing rates of the two neurons according to the reinforcements, while the firing rates of the other neurons remained unchanged. Changing the type of reinforcement during the simulation from positive to negative for neuron A and from negative to positive for neuron B resulted in a corresponding shift in their firing rate change in the direction of the reinforcement.

**Figure 5:**
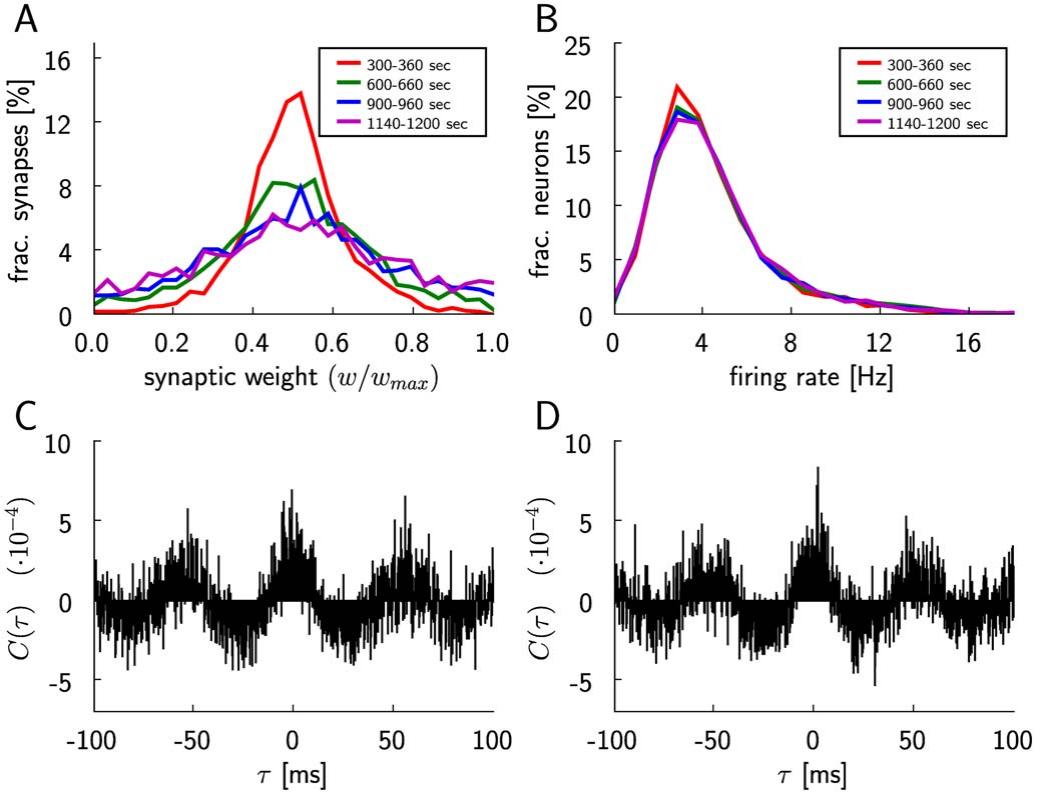
Evolution of the dynamics of a recurrent network of 4000 LIF neurons during application of reward-modulated STDP. (A) Distribution of the synaptic weights of excitatory synapses to 50 randomly chosen non-reinforced neurons, plotted for 4 different periods of simulated biological time during the simulation. The weights are averaged over 10 samples within these periods. The colors of the curves and the corresponding intervals are as follows: red (300–360 sec), green (600–660 sec), blue (900–960 sec), magenta (1140–1200 sec). (B) The distribution of average firing rates of the non-reinforced excitatory neurons in the circuit, plotted for the same time periods as in (A). The colors of the curves are the same as in (A). The distribution of the firing rates of the neurons in the circuit remains unchanged during the simulation, which covers 20 minutes of biological time. (C) Cross-correlogram of the spiking activity in the circuit, averaged over 200 pairs of non-reinforced neurons and over 60 s, with a bin size of 0.2 ms, for the period between 300 and 360 seconds of simulated biological time. It is calculated as the cross-covariance divided by the square root of the product of variances. (D) As in (C), but between seconds 1140 and 1200. (Separate plots of (B), (C), and (D) for two types of excitatory neurons that received different amounts of noise currents are given in Figure S1 and Figure S2.)

The dynamics of a network where STDP is applied to all synapses between excitatory neurons is quite sensitive to the specific choice of the STDP-rule. The preceding theoretical analysis (see Equations 10 and 11) predicts that reward-modulated STDP affects in the long run only those excitatory synapses where the firing of the postsynaptic neuron is correlated with the reward signal. In other words: the reward signal gates the effect of STDP in a recurrent network, and thereby can keep the network within a given dynamic regime. This prediction is confirmed qualitatively by the two panels of Figure 4A, which show that even after all excitatory synapses in the recurrent network have been subject to 20 minutes (in simulated biological time) of reward-modulated STDP, the network stays within the asynchronous irregular firing regime. It is also confirmed quantitatively through Figure 5. These figures show results for the simple additive version of STDP (according to Equation 3). Very similar results (see Figure S3 and Figure S4) arise from an application of the more complex STDP-rule proposed in [22] where the weight-change depends on the current weight value.

### Rewarding Spike-Times

The preceding model for the biofeedback experiment of Fetz and Baker focused on learning of firing rates. In order to explore the capabilities and limitations of reward-modulated STDP in contexts where the temporal structure of spike trains matters, we investigated another reinforcement learning scenario where a neuron should learn to respond with particular temporal spike patterns. We first apply analytical methods to derive conditions under which a neuron subject to reward-modulated STDP can achieve this.

**Figure 6:**
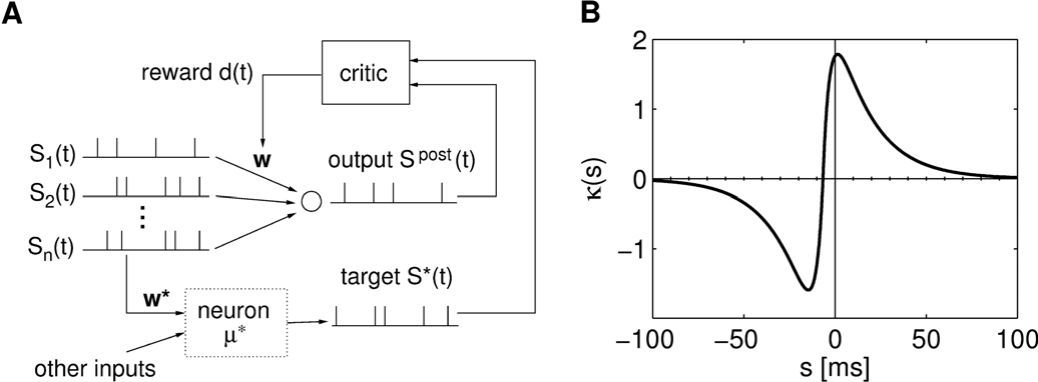
Setup for reinforcement learning of spike times. (A) Architecture. The trained neuron receives *n* input spike trains. The neuron μ* receives the same inputs plus additional inputs not accessible to the trained neuron. The reward is determined by the timing differences between the action potentials of the trained neuron and the neuron μ*. (B) A reward kernel with optimal offset from the origin of *t_κ_* = −6.6 ms. The optimal offset for this kernel was calculated with respect to the parameters from computer simulation 1 in Table 1. Reward is positive if the neuron spikes around the target spike or somewhat later, and negative if the neuron spikes much too early.

In this model, the reward signal *d*(*t*) is given in dependence on how well the output spike train 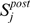 of a neuron *j* matches some rather arbitrary spike train S* (which might for example represent spike output from some other brain structure during a developmental phase). S* is produced by a neuron μ* that receives the same *n* input spike trains *S*_1_,…,*S_n_* as the trained neuron *j*, with some arbitrarily chosen weights 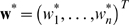, 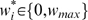. But in addition the neuron μ* receives *n*′−*n* further spike trains *S_n_*_+1_,…,*S_n_*_′_ with weights 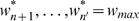. The setup is illustrated in Figure 6A. It provides a generic reinforcement learning scenario, when a quite arbitrary (and not perfectly realizable) spike output is reinforced, but simultaneously the performance of the learner can be evaluated clearly according to how well its weights *w_j_*_1_,…,*w_jn_* match those of the neuron μ* for those *n* input spike trains which both of them have in common. The reward *d*(*t*) at time *t* depends in this task on both the timing of action potentials of the trained neuron and spike times in the target spike train S*
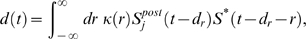
(12)where the function *κ*(*r*) with 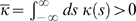 describes how the reward signal depends on the time difference *r* between a postsynaptic spike and a target spike, and *d_r_*>0 is the delay of the reward.

Our theoretical analysis (see Methods) predicts that under the assumption of constant-rate uncorrelated Poisson input statistics this reinforcement learning task can be solved by reward-modulated STDP for arbitrary initial weights if three constraints are fulfilled:
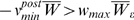
(13)

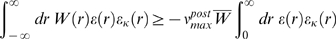
(14)

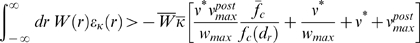
(15)The following parameters occur in these equations: ν* is the output rate of neuron μ*, 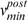 is the minimal output rate, 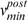 is the maximal output rate of the trained neuron, 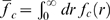 is the integral over the eligibility trace, 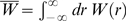 is the integral over the STDP learning curve (see Equation 2), 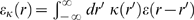 is the convolution of the reward kernel with the shape of the postsynaptic potential (PSP) *ε*(*s*), and 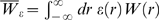 is the integral over the PSP weighted by the learning window.

If these inequalities are fulfilled and input rates are larger than zero, then the weight vector of the trained neuron converges on average from any initial weight vector to w* (i.e., it mimics the weight distribution of neuron μ* for those *n* inputs which both have in common). To get an intuitive understanding of these inequalities, we first examine the idea behind Constraint 13. This constraint assures that weights of synapses *i* with 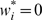 decay to zero in expectation. First note that input spikes from a spike train *S_i_* with 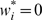 have no influence on the target spike train S*. In the linear Poisson neuron model, this leads to weight changes similar to STDP which can be described by two terms. First, all synapses are subject to depression stemming from the negative part of the learning curve *W* and random pre-post spike pairs. This weight change is bounded from below by 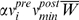 for some positive constant *α*. On the other hand, the positive influence of input spikes on postsynaptic firing leads to potentiation of the synapse bounded from above by 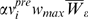. Hence the weight decays to zero if 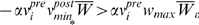, leading to Inequality 13. For synapses *i* with 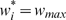, there is an additional drive, since each presynaptic spike increases the probability of a closely following spike in the target spike train S*. Therefore, the probability of a delayed reward signal after a presynaptic spike is larger. This additional drive leads to positive weight changes if Inequalities 14 and 15 are fulfilled (see Methods).

Note that also for the learning of spike times spontaneous spikes (which might be regarded as “noise”) are important, since they may lead to reward signals that can be exploited by the learning rule. It is obvious that in reward-modulated STDP, a silent neuron cannot recover from its silent state, since there will be no spikes which can drive STDP. But in addition, Condition 13 shows that in this learning scenario, the minimal output rate 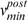
—which increases with increasing noise—has to be larger than some positive constant, such that depression is strong enough to weaken synapses if needed. On the other hand, if the noise is too strong also synapses *i* with *w_i_* = *w_max_* will be depressed and may not converge correctly. This can happen when the increased noise leads to a maximal postsynaptic rate 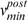 such that Constraints 14 and 15 are not satisfied anymore.

Conditions 13–15 also reveal how parameters of the model influence the applicability of this setup. For example, the eligibility trace enters the equations only in the form of its integral and its value at the reward delay in Equation 15. In fact, the exact shape of the eligibility trace is not important. The important property of an ideal eligibility trace is that it is high at the reward delay and low at other times as expressed by the fraction in Condition 15. Interestingly, the formulas also show that one has quite some freedom in choosing the form of the STDP window, as long as the reward kernel *ε_κ_* is adjusted accordingly. For example, instead of a standard STDP learning window *W* with *W*(*r*)≥0 for *r*>0 and *W*(*r*)≤0 for *r*<0 and a corresponding reward kernel *κ*, one can use a reversed learning window *W*′ defined by *W*′(*r*)≡*W*(−*r*) and a reward kernel *κ*′ such that *ε_κ_*_′_(*r*) = *ε_κ_*(−*r*). If Condition 15 is satisfied for *W* and *κ*, then it is also satisfied for *W*′ and *κ′* (and in most cases also Condition 14 will be satisfied). This reflects the fact that in reward modulated STDP the learning window defines the weight changes in combination with the reward signal.

For a given STDP learning window, the analysis reveals what reward kernels *κ* are suitable for this learning setup. From Condition 15, we can deduce that the integral over *κ* should be small (but positive), whereas the integral 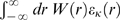 should be large. Hence, for a standard STDP learning window *W* with *W*(*r*)≥0 for *r*>0 and *W*(*r*)≤0 for *r*<0, the convolution *ε_κ_*(*r*) of the reward kernel with the PSP should be positive for *r*>0 and negative for *r*<0. In the computer simulation we used a simple kernel depicted in Figure 6B, which satisfies the aforementioned constraints. It consists of two double-exponential functions, one positive and one negative, with a zero crossing at some offset *t_κ_* from the origin. The optimal offset *t_κ_* is always negative and in the order of several milliseconds for usual PSP-shapes *ε*. We conclude that for successful learning in this scenario, a positive reward should be produced if the neuron spikes around the target spike or somewhat later, and a negative reward should be produced if the neuron spikes much too early.

### Computer Simulation 2: Learning Spike Times

In order to explore this learning scenario in a biologically more realistic setting, we trained a LIF neuron with conductance based synapses exhibiting short term facilitation and depression. The trained neuron and the neuron μ* which produced the target spike train S* both received inputs from 100 input neurons emitting spikes from a constant rate Poisson process of 15 Hz. The synapses to the trained neuron were subject to reward-modulated STDP. The weights of neuron μ* were set to 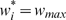 for 0≤*i*<50 and 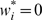 for 50≤*i*<100. In order to simulate a non-realizable target response, neuron μ* received 10 additional synaptic inputs (with weights set to *w_max_*/2). During the simulations we observed a firing rate of 18.2 Hz for the trained neuron, and 25.2 Hz for the neuron μ*. The simulations were run for 2 hours simulated biological time.

**Figure 7:**
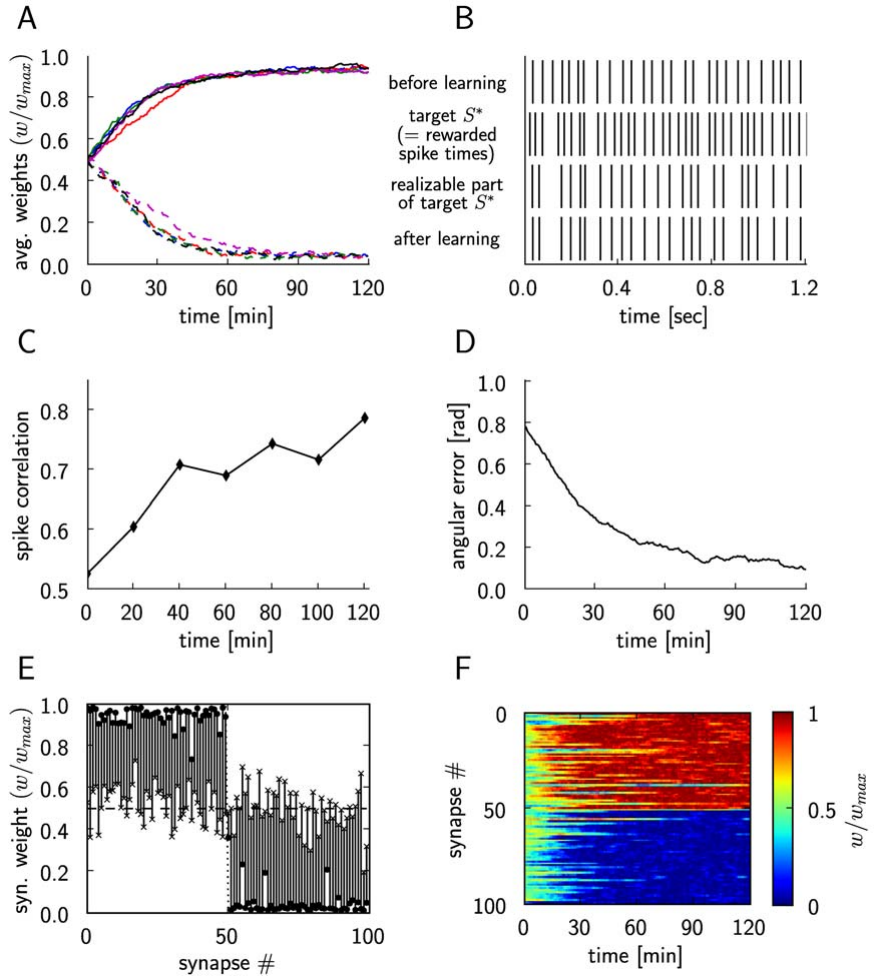
Results for reinforcement learning of exact spike times through reward-modulated STDP. (A) Synaptic weight changes of the trained LIF neuron, for 5 different runs of the experiment. The curves show the average of the synaptic weights that should converge to 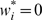 (dashed lines), and the average of the synaptic weights that should converge to 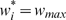 (solid lines) with different colors for each simulation run. (B) Comparison of the output of the trained neuron before (top trace) and after learning (bottom trace). The same input spike trains and the same noise inputs were used before and after training for 2 hours. The second trace from above shows those spike times S* which are rewarded, the third trace shows the realizable part of S* (i.e. those spikes which the trained neuron could potentially learn to reproduce, since the neuron μ* produces them without its 10 extra spike inputs). The close match between the third and fourth trace shows that the trained neuron performs very well. (C) Evolution of the spike correlation between the spike train of the trained neuron and the realizable part of the target spike train S*. (D) The angle between the weight vector w of the trained neuron and the weight vector w* of the neuron μ* during the simulation, in radians. (E) Synaptic weights at the beginning of the simulation are marked with ×, and at the end of the simulation with •, for each plastic synapse of the trained neuron. (F) Evolution of the synaptic weights *w*/*w_max_* during the simulation (we had chosen 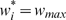 for *i*<50, 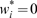 for *i*≥50).

We performed 5 repetitions of the experiment, each time with different randomly generated inputs and different initial weight values for the trained neuron. In each of the 5 runs, the average synaptic weights of synapses with 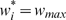 and 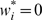 approached their target values, as shown in Figure 7A. In order to test how closely the trained neuron reproduces the target spike train S* after learning, we performed additional simulations where the same spike input was applied to the trained neuron before and after the learning. Then we compared the output of the trained neuron before and after learning with the output S* of neuron μ*. Figure 7B shows that the trained neuron approximates the part of S* which is accessible to it quite well. Figure 7C–F provide more detailed analyses of the evolution of weights during learning. The computer simulations confirmed the theoretical prediction that the neuron can learn well through reward-modulated STDP only if a certain level of noise is injected into the neuron (see preceding discussion and Figure S6).

Both the theoretical results and these computer simulations demonstrate that a neuron can learn quite well through reward-modulated STDP to respond with specific spike patterns.

### Computer Simulation 3: Testing the Analytically Derived Conditions

**Figure 8:**
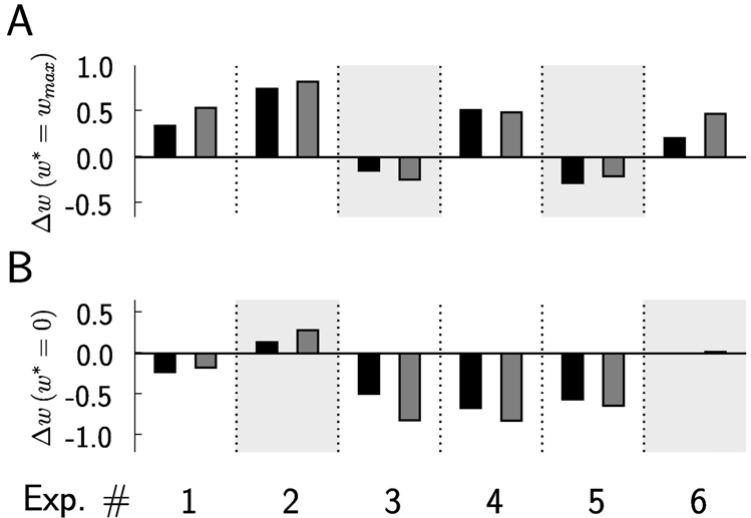
Test of the validity of the analytically derived conditions 13–15 on the relationship between parameters for successful learning with reward-modulated STDP. Predicted average weight changes (black bars) calculated from Equation 22 match in sign and magnitude the actual average weight changes (gray bars) in computer simulations, for 6 different experiments with different parameter settings (see Table 1). (A) Weight changes for synapses with 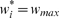. (B) Weight changes for synapses with 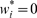. Four cases where constraints 13–15 are not fulfilled are shaded in light gray. In all of these four cases the weights move into the opposite direction, i.e., a direction that decreases rewards.

Equations 13–15 predict under which relationships between the parameters involved the learning of particular spike responses through reward-modulated STDP will be successful. We have tested these predictions by selecting 6 arbitrary settings of these parameters, which are listed in Table 1. In 4 cases (marked by light gray shading in Figure 8) these conditions were not met (either for the learning of weights with target value *w_max_*, or for the learning of weights with target value 0. Figure 8 shows that the derived learning result is not achieved in exactly these 4 cases. On the other hand, the theoretically predicted weight changes (black bar) predict in all cases the actual weight changes (gray bar) that occur for the chosen simulation times (listed in the last column of Table 1) remarkably well.

**Table 1:** Parameter values used for computer simulation 3 (see Figure 8).

Ex.	*τ_ε_* [ms]	*w_max_*	*υ^post^_min_* [Hz]	*A_+_* 10^6^	*A_−_*/*A_+_*	*τ_+_* [ms]	*A^κ^_+_*, *A^κ^_−_*	*τ^κ^*_2_ [ms]	*t_sim_* [h]
1	10	0.012	10	16.62	1.05	20	3.34, −3.12	20	5
2	7	0.020	5	11.08	1.02	15	4.58, −4.17	16	10
3	20	0.010	6	5.54	1.10	25	1.50, −1.39	40	19
4	7	0.020	5	11.08	1.07	25	4.67, −4.17	16	13
5	10	0.015	6	20.77	1.10	25	3.75, −3.12	20	2
6	25	0.005	3	13.85	1.01	25	3.34, −3.12	20	18

### Pattern Discrimination with Reward-Modulated STDP

We examine here the question whether a neuron can learn through reward-modulated STDP to discriminate between two spike patterns *P* and *N* of its presynaptic neurons, by responding with more spikes to pattern *P* than to pattern *N*. Our analysis is based on the assumption that there exist internal rewards *d*(*t*) that could guide such pattern discrimination. This reward based learning architecture is biologically more plausible than an architecture with a supervisor which provides for each input pattern a target output and thereby directly produces the desired firing behavior of the neuron (since the question becomes then how the supervisor has learnt to produce the desired spike outputs).

We consider a neuron that receives input from *n* presynaptic neurons. A pattern *X* consists of *n* spike trains, each of time length *T*, one for each presynaptic neuron. There are two patterns, *P* and *N*, which are presented in alternation to the neuron, with some reset time between presentations. For notational simplicity, we assume that each of the *n* presynaptic spike trains consists of exactly one spike. Hence, each pattern can be defined by a list of spike times: 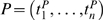, 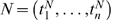, where 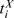 is the time when presynaptic neuron *i* spikes for pattern *X*∈{*P*,*N*}. A generalization to the easier case of learning to discriminate spatio-temporal presynaptic firing patterns (where some presynaptic neurons produce different numbers of spikes in different patterns) is straightforward, however the main characteristics of the learning dynamics are better accessible in this conceptually simpler setup. It had already been shown in [12] that neurons can learn through reward-modulated STDP to discriminate between different *spatial* presynaptic firing patterns. But in the light of the analysis of [27] it is still open whether neurons can learn with simple forms of reward-modulated STDP, such as the one considered in this article, to discriminate *temporal* presynaptic firing patterns.

We assume that the reward signal *d*(*t*) rewards—after some delay *d_r_*—action potentials of the trained neuron if pattern *P* was presented, and punishes action potentials of the neuron if pattern *N* was presented. More precisely, we assume that
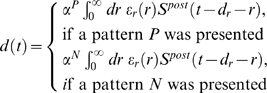
(16)with some reward kernel *ε_r_* and constants *α^N^*<0<*α^P^*. The goal of this learning task is to produce many output spikes for pattern *P*, and few or no spikes for pattern *N*.

The main result of our analysis is an estimate of the expected weight change of synapse *i* of the trained neuron for the presentation of pattern *P*, followed after a sufficiently long time *T*′ by a presentation of pattern *N*

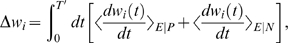
where 〈·〉*_E_*_|*X*_ is the expectation over the ensemble given that pattern *X* was presented. This weight change can be estimated as (see Methods)
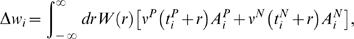
(17)where *ν^X^*(*t*) is the postsynaptic rate at time *t* for pattern *X*, and the constants 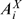 for *X*∈{*P*,*N*} are given by
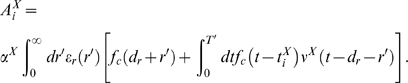
(18)As we will see shortly, an interesting learning effect is achieved if 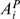 is positive and 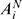 is negative. Since *f_c_*(*r*) is non-negative, a natural way to achieve this is to choose a positive reward kernel *ε_r_*(*r*)≥0 for *r*>0 and *ε_r_*(*r*) = 0 for *r*<0 (also, *f_c_*(*r*) and *ε_r_*(*r*) must not be identical to zero for all *r*).

We use Equation 17 to provide insight on when and how the classification of temporal spike patterns can be learnt with reward-modulated STDP. Assume for the moment that 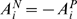. We first note that it is impossible to achieve through any synaptic plasticity rule that the time integral over the membrane potential of the trained neuron has after training a larger value for input pattern *P* than for input pattern *N*. The reason is that each presynaptic neuron emits the same number of spikes in both patterns (namely one spike). This simple fact implies that it is impossible to train a linear Poisson neuron (with any learning method) to respond to pattern *P* with more spikes than to pattern *N*. But Equation 17 implies that reward-modulated STDP increases the variance of the membrane potential for pattern *P*, and reduces the variance for pattern *N*. This can be seen as follows. Because of the specific form of the STDP learning curve *W*(*r*), which is positive for (small) positive *r*, negative for (small) negative *r*, and zero for large *r*, 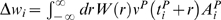 has a potentiating effect on synapse *i* if the postsynaptic rate for pattern *P* is larger (because of a higher membrane potential) shortly after the presynaptic spike at this synapse *i* than before that spike. This tends to further increase the membrane potential after that spike. On the other hand, since 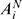 is negative, the same situation for pattern *N* has a depressing effect on synapse *i*, which counteracts the increased membrane potential after the presynaptic spike. Dually, if the postsynaptic rate shortly after the presynaptic spike at synapse *i* is lower than shortly before that spike, the effect on synapse *i* is depressing for pattern *P*. This leads to a further decrease of the membrane potential after that spike. In the same situation for pattern *N*, the effect is potentiating, again counteracting the variation of the membrane potential. The total effect on the postsynaptic membrane potential is that the fluctuations for pattern *P* are increased, while the membrane potential for pattern *N* is flattened.

For the LIF neuron model, and most reasonable other non-linear spiking neuron models, as well as for biological neurons in-vivo and in-vitro [28]–[30], larger fluctuations of the membrane potential lead to more action potentials. As a result, reward-modulated STDP tends to increase the number of spikes for pattern *P* for these neuron models, while it tends to decrease the number of spikes for pattern *N*, thereby enabling a discrimination of these purely temporal presynaptic spike patterns.

### Computer Simulation 4: Learning Pattern Classification

We tested these theoretical predictions through computer simulations of a LIF neuron with conductance based synapses exhibiting short-term depression and facilitation. Both patterns, *P* and *N*, had 200 input channels, with 1 spike per channel (hence this is the extreme where *all* information lies in the timing of presynaptic spikes). The spike times were drawn from an uniform distribution over a time interval of 500 ms, which was the duration of the patterns. We performed 1000 training trials where the patterns *P* and *N* were presented to the neuron in alternation. To introduce exploration for this reinforcement learning task, the neuron had injected 20% of the Ornstein-Uhlenbeck process conductance noise (see Methods for further details).

**Figure 9:**
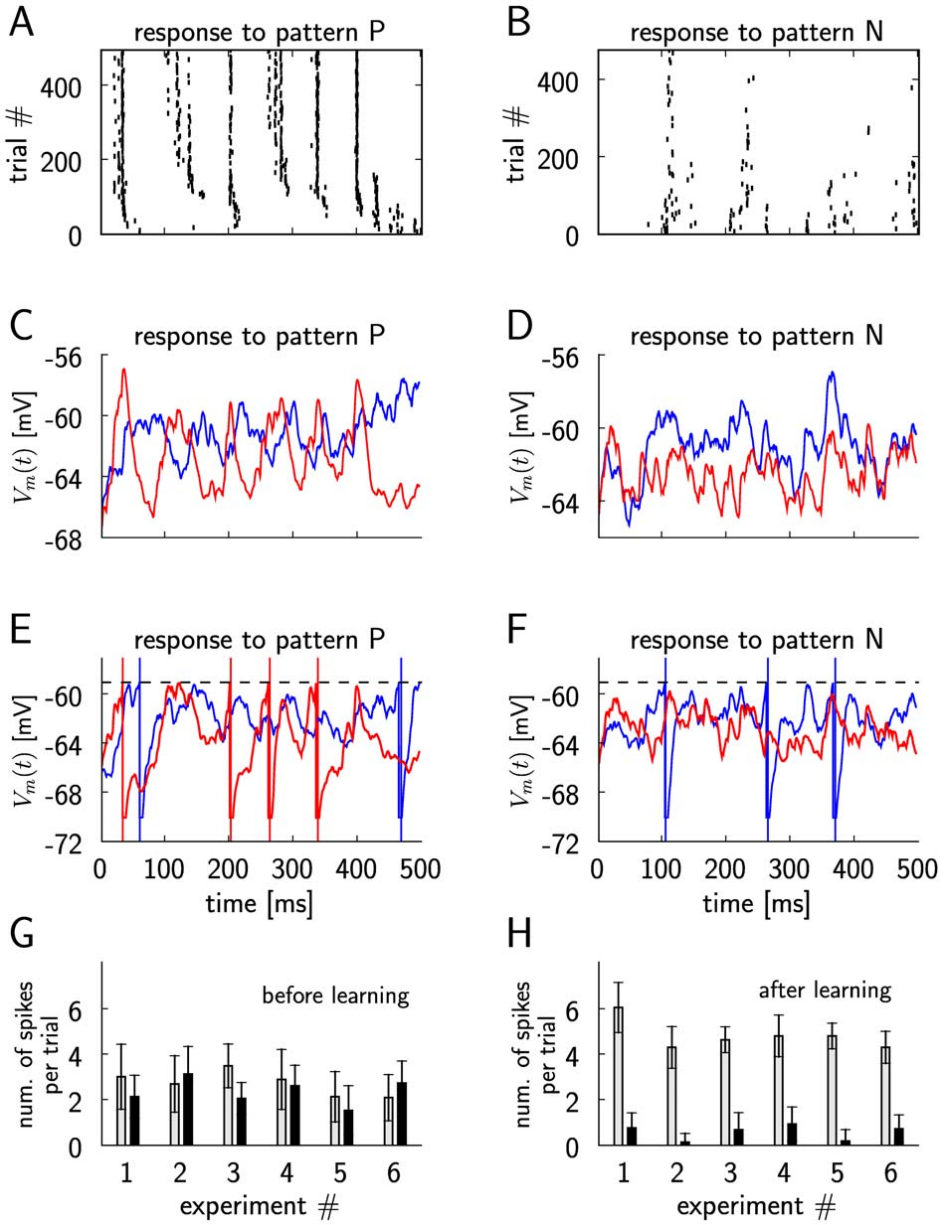
Training a LIF neuron to classify purely temporal presynaptic firing patterns: a positive reward is given for firing of the neuron in response to a temporal presynaptic firing pattern *P*, and a negative reward for firing in response to another temporal pattern *N*. (A) The spike response of the neuron for individual trials, during 500 training trials when pattern *P* is presented. Only the spikes from every 4-th trial are plotted. (B) As in (A), but in response to pattern *N*. (C) The membrane potential *V_m_*(*t*) of the neuron during a trial where pattern *P* is presented, before (blue curve) and after training (red curve), with the firing threshold removed. The variance of the membrane potential increases during learning, as predicted by the theory. (D) As in (C), but for pattern *N*. The variance of the membrane potential for pattern *N* decreases during learning, as predicted by the theory. (E) The membrane potential *V_m_*(*t*) of the neuron (including action potentials) during a trial where pattern *P* is presented before (blue curve) and after training (red curve). The number of spikes increases. (F) As in (E), but for trials where pattern *N* is given as input. The number of spikes decreases. (G) Average number of output spikes per trial before learning, in response to pattern *P* (gray bars) and pattern *N* (black bars), for 6 experiments with different randomly generated patterns *P* and *N*, and different random initial synaptic weights of the neuron. (H) As in (G), for the same experiments, but after learning. The average number of spikes per trial increases after training for pattern *P*, and decreases for pattern *N*.

The theoretical analysis predicted that the membrane potential will have after learning a higher variance for pattern *P*, and a lower variance for pattern *N*. When in our simulation of a LIF neuron the firing of the neuron was switched off (by setting the firing threshold potential too high) we could observe the membrane potential fluctuations undisturbed by the reset mechanism after each spike (see Figure 9C and 9D). The variance of the membrane potential did in fact increase for pattern *P* from 2.49 (mV)^2^ to 5.43 (mV)^2^ (Figure 9C), and decrease for pattern *N* (Figure 9D), from 2.34 (mV)^2^ to 1.33 (mV)^2^. The corresponding plots with the firing threshold included are given in panels E and F, showing an increased member of spikes of the LIF neuron for pattern *P*, and a decreased number of spikes for pattern *N*. Furthermore, as Figure 9A and 9B show, the increased variance of the membrane potential for the positively reinforced pattern *P* led to a stable temporal firing pattern in response to pattern *P*.

We repeated the experiment 6 times, each time with different randomly generated patterns *P* and *N*, and different random initial synaptic weights of the neuron. The results in Figure 9G and 9H show that the learning of temporal pattern discrimination through reward-modulated STDP does not depend on the temporal patterns that are chosen, nor on the initial values of synaptic weights.

### Computer Simulation 5: Training a Readout Neuron with Reward-Modulated STDP To Recognize Isolated Spoken Digits

**Figure 10:**
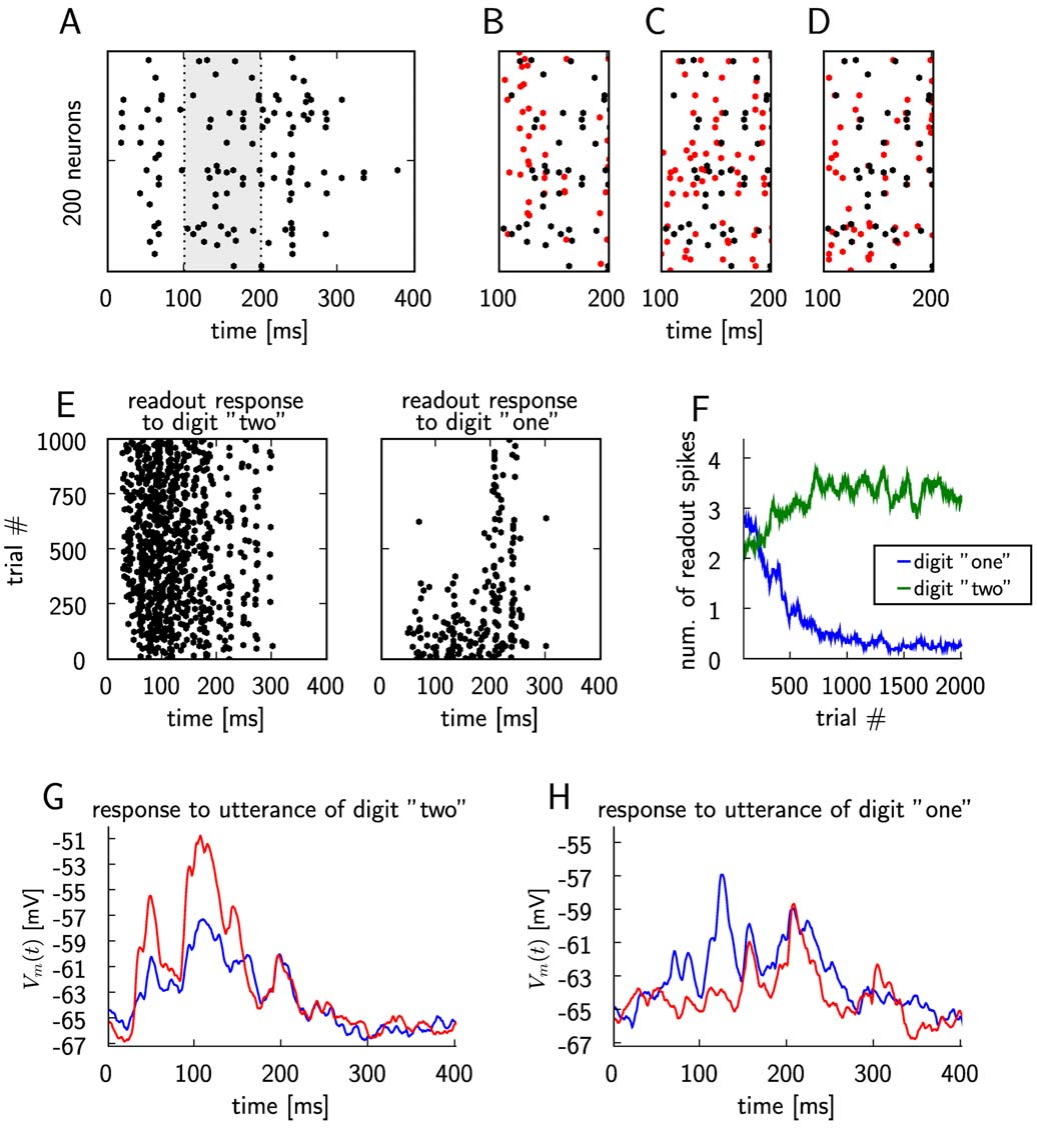
A LIF neuron is trained through reward-modulated STDP to discriminate as a “readout neuron” responses of generic cortical microcircuits to utterances of different spoken digits. (A) Circuit response to an utterance of digit “one” (spike trains of 200 out of 540 neurons in the circuit are shown). The response within the time period from 100 to 200 ms (marked in gray) is used as a reference in the subsequent 3 panels. (B) The circuit response from (A) (black) for the period between 100 and 200 ms, and the circuit response to an utterance of digit “two” (red). (C) The circuit spike response from (A) (black) and a circuit response for another utterance of digit “one” (red), also shown for the period between 100 and 200 ms. (D) The circuit spike response from (A) (black), and another circuit response to the same utterance in another trial (red). The responses differ due to the presence of noise in the circuit. (E) Spike response of the LIF readout neuron for different trials during learning, for trials where utterances of digit “two” (left plot) and digit “one” (right plot) are presented as circuit inputs. The spikes from each 4th trial are plotted. (F) Average number of spikes in the response of the readout during training, in response to digit “one” (blue) and digit “two” (green). The number of spikes were averaged over 40 trials. (G) The membrane potential *V_m_*(*t*) of the neuron during a trial where an input pattern corresponding to an utterance of digit “two” is presented, before (blue curve) and after training (red curve), with the firing threshold removed. (H) As in (G), but for an input pattern corresponding to an utterance of digit “one”. The variance of the membrane potential increases during learning for utterances of the rewarded digit, and decreases for the non-rewarded digit.

A longstanding open problem is how a biologically realistic neuron model can be trained in a biologically plausible manner to extract information from a generic cortical microcircuit. Previous work [31]–[35] has shown that quite a bit of salient information about recent and past inputs to the microcircuit can be extracted by a non-spiking linear readout neuron (i.e., a perceptron) that is trained by linear regression or margin maximization methods. Here we examine to what extent a LIF readout neuron with conductance based synapses (subject to biologically realistic short term synaptic plasticity) can learn through reward-modulated STDP to extract from the response of a simulated cortical microcircuit (consisting of 540 LIF neurons), see Figure 10A, the information which spoken digit (transformed into spike trains by a standard cochlea model) is injected into the circuit. In comparison with the preceding task in simulation 4, this task is easier because the presynaptic firing patterns that need to be discriminated differ in temporal and spatial aspects (see Figure 10B; Figure S10 and S11 show the spike trains that were injected into the circuit). But this task is on the other hand more difficult, because the circuit response (which creates the presynaptic firing pattern for the readout neuron) differs also significantly for two utterances of the same digit (Figure 10C), and even for two trials for the same utterance (Figure 10D) because of the intrinsic noise in the circuit (which was modeled according to [26] to reflect in-vivo conditions during cortical UP-states). The results shown in Figure 10E–H demonstrate that nevertheless this learning experiment was successful. On the other hand we were not able to achieve in this way speaker-independent word recognition, which had been achieved in [31] with a linear readout. Hence further work will be needed in order to clarify whether biologically more realistic models for readout neurons can be trained through reinforcement learning to reach the classification capabilities of perceptrons that are trained through supervised learning.

## Methods

We first describe the simple neuron model that we used for the theoretical analysis, and then provide derivations of the equations that were discussed in the preceding section. After that we describe the models for neurons, synapses, and synaptic background activity (“noise”) that we used in the computer simulations. Finally we provide technical details to each of the 5 computer simulations that we discussed in the preceding section.

### Linear Poisson Neuron Model

In our theoretical analysis, we use a linear Poisson neuron model whose output spike train 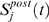 is a realization of a Poisson process with the underlying instantaneous firing rate *R_j_*(*t*). The effect of a spike of presynaptic neuron *i* at time *t*′ on the membrane potential of neuron *j* is modeled by an increase in the instantaneous firing rate by an amount *w_ji_*(*t*′)*ε*(*t*−*t*′), where *ε* is a response kernel which models the time course of a postsynaptic potential (PSP) elicited by an input spike. Since STDP according to [12] has been experimentally confirmed only for excitatory synapses, we will consider plasticity only for excitatory connections and assume that *w_ji_*≥0 for all *i* and *ε*(*s*)≥0 for all *s*. Because the synaptic response is scaled by the synaptic weights, we can assume without loss of generality that the response kernel is normalized to 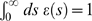. In this linear model, the contributions of all inputs are summed up linearly:
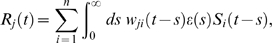
(19)where *S*_1_,…,*S_n_* are the *n* presynaptic spike trains. Since the instantaneous firing rate *R*(*t*) is analogous to the membrane potential of other neuron models, we occasionally refer to *R*(*t*) as the “membrane potential” of the neuron.

### Learning Equations

In the following, we denote by 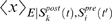 the ensemble average of a random variable *x* given that neuron *k* spikes at time *t* and neuron *i* spikes at time *t*′. We will also sometimes indicate the variables *Y*_1_,*Y*_2_,… over which the average of *x* is taken by writing 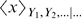.

#### Derivation of Equation 6

Using Equations 5, 1, and 4, we obtain the expected weight change between time *t* and *t*+*T*

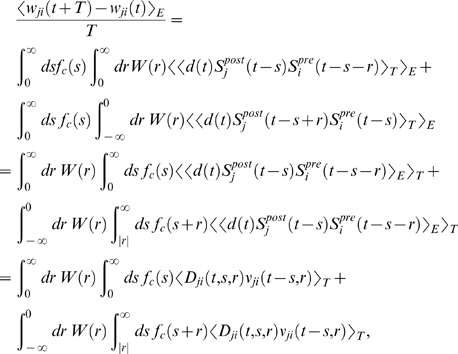
with *D_ji_*(*t*,*s*,*r*) = 〈*d*(*t*)|Neuron *j* spikes at *t*−*s*, and neuron *i* spikes at *t*−*s*−*r*〉*_E_*, and the joint firing rate *ν_ji_*(*t*,*r*) = 〈*S_j_*(*t*)*S_i_*(*t*−*r*)〉*_E_* describes correlations between spike timings of neurons *j* and *i*. The joint firing rate *ν_ji_*(*t*−*s*,*r*) depends on the weight at time *t*−*s*. If the learning rate defined by the magnitude of *W*(*r*) is small, the synaptic weights can be assumed constant on the time scale of *T*. Thus, the time scales of neuronal dynamics are separated from the slow time scale of learning. For slow learning, synaptic weights integrate a large number of small changes. We can then expect that averaged quantities enter the learning dynamics. In this case, we can argue that fluctuations of a weight *w_ji_* about its mean are negligible and it can well be approximated by its average 〈*w_ji_*〉*_E_* (it is “self-averaging”, see [21],[36]). To ensure that average quantities enter the learning dynamics, many presynaptic and postsynaptic spikes as well as many independently delivered rewards at varying delays have to occur within *T*. Hence, in general, the time scale of single spike occurrences and the time scale of the eligibility trace is required to be much smaller than the time scale of learning. If time scales can be separated, we can drop the expectation on the left hand side of the last equation and write
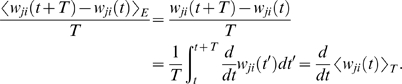
We thus obtain Equation 6:
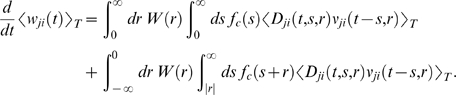



#### Simplification of Equation 6

In order to simplify this equation, we first observe that *W*(*r*) is vanishing for large |*r*|. Hence we can approximate the integral over the learning window by a bounded integral 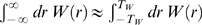 for some *T_W_*>0 and *T_W_*≪*T*. In the analyzes of this article, we consider the case where reward is delivered with a relatively large temporal delay. To be more precise, we assume that a pre-post spike pair has an effect on the reward signal only after some minimal delay *d_r_* and that we can write 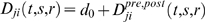 for some baseline reward *d*_0_ and a part which depends on the timing of pre-post spike pairs with 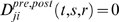 for *s*<*d_r_* and *d_r_*>*T_W_*. We can then approximate the second term of Equation 6:
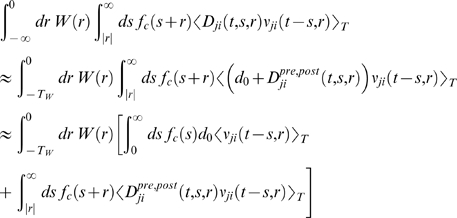
because 〈*ν_ji_*(*t*−*s*−*r*,*r*)〉*_T_*≈〈*ν_ji_*(*t*−*s*,*r*)〉*_T_* for *r*∈[−*T_W_*,*T_W_*] and *T_W_*≪*T*. Since 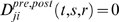 for *s*≤*T_W_*, the second term in the brackets is equivalent to 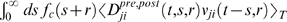 which in turn is approximately given by 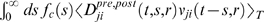 if we assume that *f_c_*(*s*+*r*)≈*f_c_*(*s*) for *s*≥*d_r_* and |*r*|<*T_W_*. We can thus approximate the second term of Equation 6 as
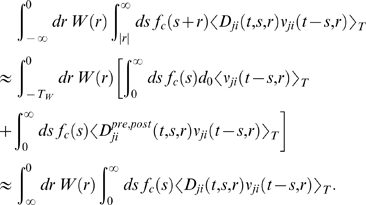
With this approximation, the first and second term of Equation 6 can be combined in a single integral to obtain Equation 8.

### Derivations for the Biofeedback Experiment

We assume that a reward with the functional form *ε_r_* is delivered for each postsynaptic spike with a delay *d_r_*. The reward as time *t* is therefore
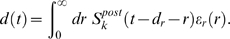



### Weight change for the reinforced neuron (derivation of Equation 10)

The reward correlation for a synapse *ki* afferent to the reinforced neuron is 
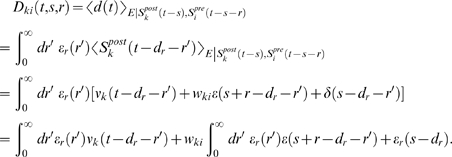
If we assume that the output firing rate is constant on the time scale of the reward function, the first term vanishes. We rewrite the result as
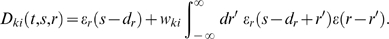
The mean weight change for weights to the reinforced neuron is therefore
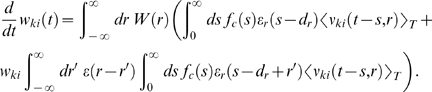
(20)We show that the second term in the brackets is very small compared to the first term:
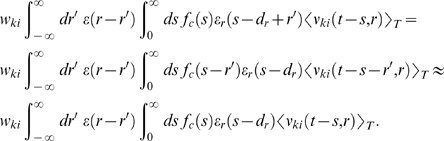
The last approximation is based on the assumption that *f_c_*(*s*)≈*f_c_*(*s*−*r*′) and 〈*ν_ki_*(*t*−*r*′,*r*)〉*_T_*≈〈*ν_ki_*(*t*,*r*)〉*_T_* for *r*′∈[−*T_W_*−*T_ε_*,*T_W_*]. Here, *T_W_* is the time scale of the learning window (see above), and *T_ε_* is time scale of the PSP, i.e., we have *ε*(*s*)≈0 for *s*≥*T_ε_*. Since 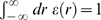 by definition, we see that this is the first term in the brackets of Equation 20 scaled by *w_ki_*. For neurons with many input synapses we have *w_ki_*≪1. Thus the second term in the brackets of Equation 20 is small compared to the first term. We therefore have
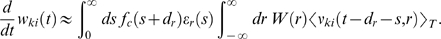



### Weight change for non-reinforced neurons (derivation of Equation 11)

The reward correlation of a synapse *ji* to a non-reinforced neuron *j* is given by
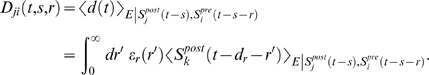
We have
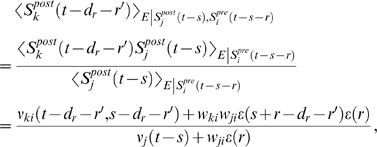
for which we obtain
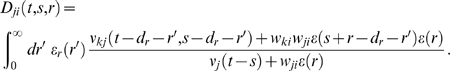
In analogy to the previous derivation, we assume here that the firing rate *ν_j_*(*t*−*s*) in the denominator results from many PSPs. Hence, the single PSP *w_ji_*ε**(*r*) is small compared to *ν_j_*(*t*−*s*). Similarly, we assume that with weights *w_ki_*, *w_ji_*≪1, the second term in the nominator is small compared to the joint firing rate *ν_kj_*(*t*−*d_r_*−*r*′,*s*−*d_r_*−*r*′). We therefore approximate the reward correlation by
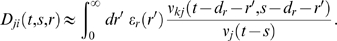
Hence, the reward correlation of a non-reinforced neuron depends on the correlation of this neuron with the reinforced neuron. The mean weight change for a non-reinforced neuron *j*≠*k* is therefore
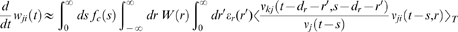
This equation deserves a remark for the case that *ν_j_*(*t*−*s*) is zero, since it appears in the denominator of the fraction. Note that in this case, both *ν_kj_*(*t*−*d_r_*−*r*′,*s*−*d_r_*−*r*′) and *ν_ji_*(*t*−*s*,*r*) are zero. In fact, if we take the limit *ν_j_*(*t*−*s*)→0, then both of these factors approach zero at least as fast. Hence, in the limit of *ν_j_*(*t*−*s*)→0, the term in the angular brackets evaluates to zero. This reflects the fact that since STDP is driven by pre- and postsynaptic spikes, there is no weight change if no postsynaptic spikes occur.

#### For uncorrelated neurons, Equation 11 evaluates to zero

For uncorrelated neurons *k*, *j*, *ν_kj_*(*t*−*d_r_*−*r*′,*s*−*d_r_*−*r*′) can be factorized into *ν_k_*(*t*−*d_r_*−*r*′)*ν_j_*(*t*−*s*), and we obtain
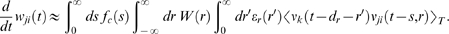
This evaluates approximately to zero if the mean output rate of neuron *k* is constant on the time scale of the reward kernel.

### Analysis of Spike-Timing-Dependent Rewards (Derivation of Conditions 13–15)

Below, we will indicate the variables *Y*_1_,*Y*_2_,… over which the average of *x* is taken by writing 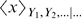. From Equation 12, we can determine the reward correlation for synapse *i*

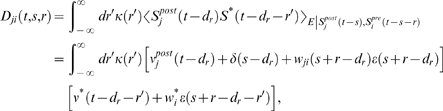
(21)where 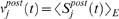 denotes the instantaneous firing rate of the trained neuron at time *t*, and *ν* ^*^(*t*) = 〈*S* ^*^(*t*)〉*_E_* denotes the instantaneous rate of the target spike train at time *t*. Since weights are changing very slowly, we have *w_ji_*(*t*−*s*−*r*)≈*w_ji_*(*t*). In the following, we will drop the dependence of *w_ji_* on *t* for brevity. For simplicity, we assume that input rates are stationary and uncorrelated. In this case (since the weights are changing slowly), also the correlations between inputs and outputs can be assumed stationary, *ν_ji_*(*t*,*r*) = *ν_ji_*(*r*). With constant input rates, we can rewrite Equation 21 as
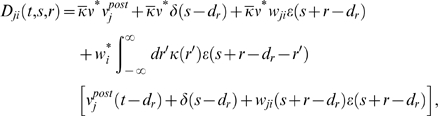
with 
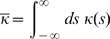
. We use this results to obtain the temporally smoothed weight change for synapse *ji*. With stationary correlations, we can drop the dependence of *ν_ji_* on *t* and write *ν_ji_*(*t*,*r*) = *ν_ji_*(*r*). Furthermore, we define 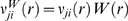 and obtain
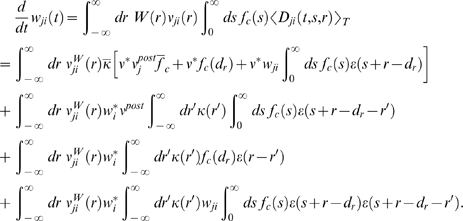
We assume that the eligibility function *f_c_*(*d_r_*)≈*f_c_*(*d_r_*+*r*) if |*r*| is on the time scale of a PSP, the learning window, or the reward kernel, and that *d_r_* is large compared to these time scales. Then, we have
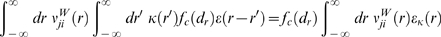
where 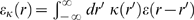 is the convolution of the reward kernel with the PSP. Furthermore, we find
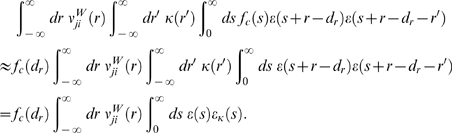
With these simplifications, and the abbreviation 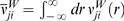 we obtain the weight change at synapse *ji*

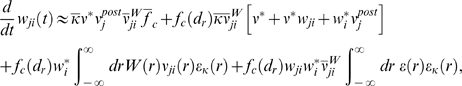
where 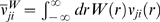.

For uncorrelated Poisson input spike trains of rate 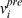 and the linear Poisson neuron model, the input-output correlations are 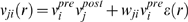. With these correlations, we obtain 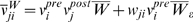 where 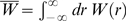, and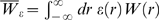. The weight change at synapse *ji* is then
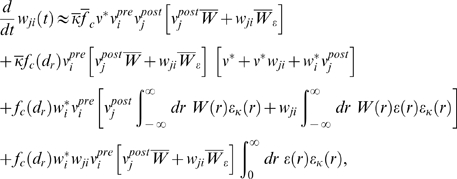
(22)


We will now bound the expected weight change for synapses *ji* with 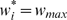 and for synapses *jk* with 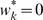. In this way we can derive conditions for which the expected weight change for the former synapses is positive, and that for the latter type is negative. First, we assume that the integral over the reward kernel is positive. In this case, the weight change given by Equation 22 is negative for synapses *i* with 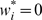 if and only if 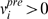, and 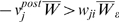. In the worst case, *w_ji_* is *w_max_* and 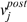 is small. We have to guarantee some minimal output rate 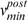 such that even if *w_ji_* = *w_max_*, this inequality is fulfilled. This could be guaranteed by some noise current. Given such minimal output rate, we can state the first inequality which guarantees convergence of weights *w_ji_* with 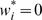


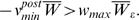
For synapses *ji* with 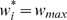, we obtain two more conditions. The approximate weight change is given by
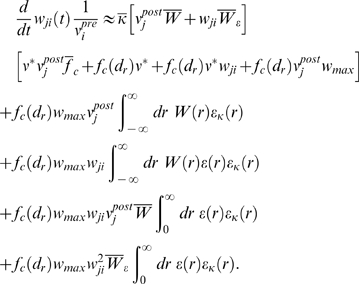
The last term in this equation is positive and small. We can ignore it in our sufficient condition. The second to last term is negative. We will include in our condition that the third to last term compensates for this negative term. Hence, the second condition is
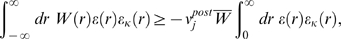
which should be satisfied in most setups. If we assume that this holds, we obtain
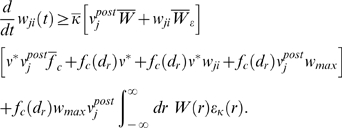
which should be positive. We obtain the following inequality
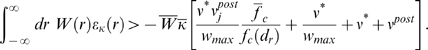
All three inequalities are summarized in the following:
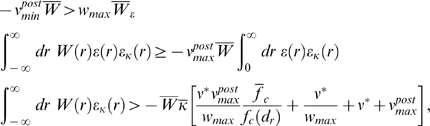
where 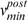 is the maximal output rate. If these inequalities are fulfilled and input rates are positive, then the weight vector converges on average from any initial weight vector to w*. The second condition is less severe, and should be easily fulfilled in most setups. If this is the case, the first Condition 13 ensures that weights with w* = 0 are depressed while the third Condition 15 ensures that weights with w* = *w_max_* are potentiated.

### Analysis of the Pattern Discrimination Task (Derivation of Equation 17)

We assume that a trial consists of the presentation of a single pattern starting at time *t* = 0. We compute the weight change for a single trial given that pattern *X*∈{*P*,*N*} was presented with the help of Equations 1, 3, and 4 as
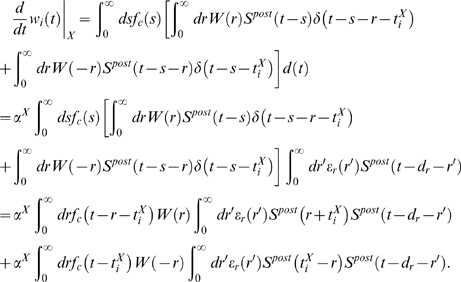
We can compute the average weight change given that pattern *X* was presented:
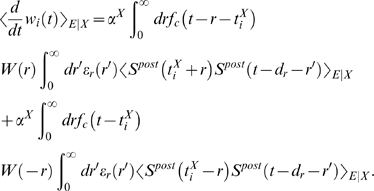
If we assume that *f_c_* is approximately constant on the time scale of the learning window *W*, we can simplify this to
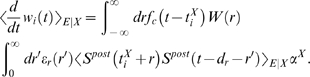
For the linear Poisson neuron, we can write the auto-correlation function as
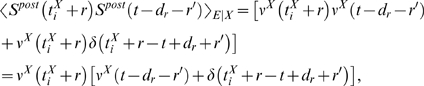
where *ν^X^*(*t*) = 〈*S^post^*(*t*)〉*_E_*_|*X*_ is the ensemble average rate at time *t* given that pattern *X* was presented. If an experiment for a single pattern runs over the time interval [0,*T*′], we can compute the total average weight change 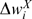 of a trial given that pattern *X* was presented as
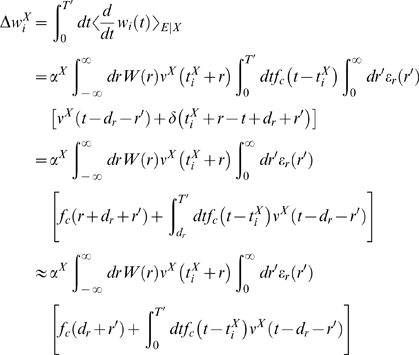
(23)By defining
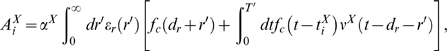
we can write Equation 23 as
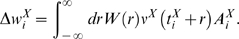
We assume that eligibility traces and reward signals have settled to zero before a new pattern is presented. The expected weight change for the successive presentation of both patterns is therefore
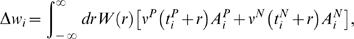
The equations can easily be generalized to the case where multiple input spikes per synapse are allowed and where jitter on the templates is allowed. However, the main effect of the rule can be read off the equations given here.

### Common Models and Parameters of the Computer Simulations

We describe here the models and parameter values that were used in all our computer simulations. We will specify in a subsequent section the values of other parameters that had to be chosen differently in individual computer simulations, in dependence of their different setups and requirements of each computer simulation.

### LIF Neuron Model

For the computer simulations LIF neurons with conductance-based synapses were used. The membrane potential *V_m_*(*t*) of this neuron model is given by:
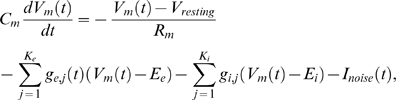
(24)where *C_m_* is the membrane capacitance, *R_m_* is the membrane resistance, *V_resting_* is the resting potential, and *g_e_*_,*j*_(*t*) and *g_i_*_,*j*_(*t*) are the *K_e_* and *K_i_* synaptic conductances from the excitatory and inhibitory synapses respectively. The constants *E_e_* and *E_i_* are the reversal potentials of excitatory and inhibitory synapses. *I_noise_* represents the synaptic background current which the neuron receives (see below for details).

Whenever the membrane potential reaches a threshold value *V_thresh_*, the neuron produces a spike, and its membrane potential is reset to the value of the reset potential *V_reset_*. After a spike, there is a refractory period of length *T_refract_*, during which the membrane potential of the neuron remains equal to the value *V_m_*(*t*) = *V_reset_*. After the refractory period *V_m_*(*t*) continues to change according to Equation 24.

For a given synapse, the dynamics of the synaptic conductance *g*(*t*) is defined by
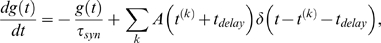
(25)where *A*(*t*) is the amplitude of the postsynaptic response (PSR) to a single presynaptic spike, which varies over time due to the inherent short-term dynamics of the synapse, and {*t* ^(*k*)^} are the spike times of the presynaptic neuron. The conductance of the synapse decreases exponentially with time constant *τ_syn_*, and increases instantaneously by amount of *A*(*t*) whenever the presynaptic neuron spikes.

In all computer simulations we used the following values for the neuron and synapse parameters. The membrane resistance of the neurons was *R_m_* = 100 MΩ, the membrane capacitance *C_m_* = 0.3 nF, the resting potential, reset potential and the initial value of the membrane potential had the same value of *V_resting_* = *V_reset_* = *V_m_*(0) = −70 mV, the threshold potential was set to *V_thresh_* = −59 mV and the refractory period *T_refract_* = 5 ms. For the synapses we used a time constant set to *τ_syn_* = 5 ms, reversal potential *E_e_* = 0 mV for the excitatory synapses and *E_e_* = −75 mV for the inhibitory synapses. All synapses had a synaptic delay of *t_delay_* = 1 ms.

### Short-Term Dynamics of Synapses

We modeled the short-term dynamics of synapses according to the phenomenological model proposed in [37], where the amplitude *A_k_* = *A*(*t_k_*+*t_delay_*) of the postsynaptic response for the *k*th spike in a spike train with inter-spike intervals Δ_1_,Δ_2_,…,Δ*_k_*_−1_ is calculated with the following equations
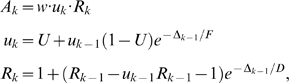
(26)with hidden dynamic variables *u*∈[0,1] and *R*∈[0,1] whose initial values for the 1st spike are *u*_1_ = *U* and *R* = 1 (see [38] for a justification of this version of the equations, which corrects a small error in [37] ). The variable *w* is the synaptic weight which scales the amplitudes of postsynaptic responses. If long-term plasticity is introduced, this variable is a function of time. In the simulations, for the neurons in the circuits the values for the U, D and F parameters were drawn from Gaussian distributions with mean values which depended on whether the type of presynaptic and postsynaptic neuron of the synapse is excitatory or inhibitory, and were chosen according to the data reported in [37] and [39]. The mean values of the Gaussian distributions are given in Table 2, and the standard deviation was chosen to be 50% of its mean. Negative values were replaced with values drawn from uniform distribution with a range between 0 and twice the mean value. For the simulations involving individual trained neurons, the U, D, and F parameters of these neurons were set to the values from Table 2.

**Table 2:** Mean values of the U, D, and F parameters in the model from [37] for the short-term dynamics of synapses, depending on the type of the presynaptic and postsynaptic neuron (excitatory or inhibitory).

Source/Dest.	Exc. (U, D, F)	Inh. (U, D, F)
Exc.	0.5, 1.1, 0.02	0.25, 0.7, 0.02
Inh.	0.05, 0.125, 1.2	0.32, 0.144, 0.06

These mean values, based on experimental data from [37],[39], were used in all computer simulations.

We have carried out control experiments with current-based synapses that were not subject to short-term plasticity (see Figure S5, Figure S8, and Figure S9; successful control experiments with static current-based synapses were also carried out for computer simulation 1, results not shown). We found that the results of all our computer simulations also hold for static current-based synapses.

### Model of Background Synaptic Activity

To reproduce the background synaptic input cortical neurons receive in vivo, the neurons in our models received an additional noise process as conductance input. The noise process we used is a point-conductance approximation model, described in [26]. According to [26], this noise process models the effect of a bombardment by a large number of synaptic inputs in vivo, which causes membrane potential depolarization, referred to as “high conductance” state. Furthermore, it was shown that it captures the spectral and amplitude characteristics of the input conductances of a detailed biophysical model of a neocortical pyramidal cell that was matched to intracellular recordings in cat parietal cortex in vivo. The ratio of average contributions of excitatory and inhibitory background conductances was chosen to be 5 in accordance to experimental studies during sensory responses (see [40]–[42]). In this model, the noisy synaptic current *I_noise_* in Equation 24 is a sum of two currents:
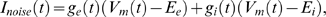
(27)where *g_e_*(*t*) and *g_i_*(*t*) are time-dependent excitatory and inhibitory conductances. The values of the respective reversal potentials were *E_e_* = 0 mV and *E_i_* = −75 mV. The conductances *g_e_*(*t*) and *g_i_*(*t*) were modeled according to [26] as a one-variable stochastic process similar to an Ornstein-Uhlenbeck process:
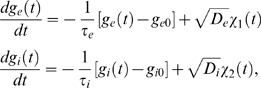
with mean *g_e_*_0_ = 0.012 *µ*S, noise-diffusion constant *D_e_* = 0.003 *µ*S and time constant *τ_e_* = 2.7 ms for the excitatory conductance, and mean *g_i_*_0_ = 0.057 *µ*S, noise-diffusion constant *D_i_* = 0.0066 *µ*S, and time constant *τ_i_* = 10.5 ms for the inhibitory conductance. *χ*_1_(*t*) and *χ*_2_(*t*) are Gaussian white noise of zero mean and unit standard deviation.

Since these processes are Gaussian stochastic processes, they can be numerically integrated by an exact update rule:
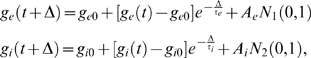
where *N*_1_(0,1) and *N*_2_(0,1) are normal random numbers (zero mean, unit standard deviation) and *A_e_*, *A_i_* are amplitude coefficients given by:
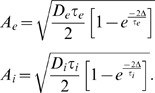



### Reward-Modulated STDP

For the computer simulations we used the following parameters for the STDP window function *W*(*r*): *A*_+_ = 0.01*w_max_*, *A*_−_/*A*_+_ = 1.05, *τ*_+_ = *τ*_−_ = 30 ms. *w_max_* denotes the hard bound of the synaptic weight of the particular plastic synapse. Note that the parameter *A*_+_ can be given arbitrary value in this plasticity rule, since it can be scaled together with the reward signal, i.e. multiplying the reward signal by some constant and dividing *A*_+_ by the same constant results in identical time evolution of the weight changes. We have set *A*_+_ to be 1% of the maximum synaptic weight.

We used the *α*-function to model the eligibility trace kernel *f_c_*(*t*)
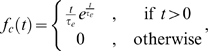
(28)where the time constant *τ_e_* was set to *τ_e_* = 0.4 s in all computer simulations.

For computer simulations 1 and 4 we performed control experiments (see Figure S3, Figure S4, and Figure S7) with the weight-dependent synaptic update rule proposed in [22], instead of the purely additive rule in Equation 3. We used the parameters proposed in [22], i.e. *μ* = 0.4, *α* = 0.11, *τ*_+_ = *τ*_−_ = 20 ms. The *w*_0_ parameter was calculated according to the formula: 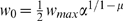 where *w_max_* is the maximum synaptic weight of the synapse. 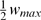 is equal to the initial synaptic weight for the circuit neurons, or to the mean of the distribution of the initial weights for the trained neurons.

### Initial Weights of Trained Neurons

The synaptic weights of excitatory synapses to the trained neurons in experiments 2–5 were initialized from a Gaussian distribution with mean *w_max_*/2. The standard deviation was set to *w_max_*/10 bounded within the range [3*w_max_*/10,7*w_max_*/10].

### Software

All computer simulations were carried out with the PCSIM software package (http://www.lsm.tugraz.at/pcsim). PCSIM is a parallel simulator for biologically realistic neural networks with a fast c++ simulation core and a Python interface. It has been developed by Thomas Natschläger and Dejan Pecevski. The time step of simulation was set to 0.1 ms.

### Details to Individual Computer Simulations

For all computer simulations, both for the cortical microcircuits and readout neurons, the same parameters values for the neuron and synapse models and the reward-modulated STDP rule were used, as specified in the previous section (except in computer simulation 3, where the goal was to test the theoretical predictions for different values of the parameters). Each of the computer simulations in this article modeled a specific task or experimental finding. Consequently, the dependence of the reward signal on the behavior of the system had to be modeled in a specific way for each simulation (a more detailed discussion of the reward signal can be found in the Discussion section). The parameters for that are given below in separate subsections which address the individual simulations. Furthermore, some of the remaining parameters in the experiments, i.e. the values of the synaptic weights, the number of synapses of a neuron, number of neurons in the circuit and the Ornstein-Uhlenbeck (OU) noise levels were chosen to achieve different goals depending on the particular experiment. Briefly stated, these values were tuned to achieve a certain level of firing activity in the neurons, a suitable dynamical regime of the activity in the circuits, and a specific ratio between amount of input the neurons receive from the input synapses and the input generated by the noise process.

We carried out two types of simulations: simulations of cortical microcircuits in computer simulations 1 and 5, and training of readout neurons in computer simulations 2, 3, 4, and 5. In the following we discuss these two types of simulations in more detail.

### Cortical Microcircuits

The values of the initial weights of the excitatory and inhibitory synapses for the cortical microcircuits are given in Table 3. All synaptic weights were bounded in the range between 0 and twice the initial synaptic weight of the synapse.

**Table 3:** Specific parameter values for the cortical microcircuits in computer simulation 1 and 5.

Simulation No.	Neurons	*p_ee_*, *p_ei_*, *p_ie_*, *p_ii_*	*w_exc_*(0) [nS]	*w_inh_* [nS]	*C_OU_*
1	4000	0.02,0.02,0.024,0.016	10.7	211.6	1.0, 0.2
5	540	0.1	0.784	5.1	0.4

*p_conn_* is the connection probability, *w_exc_*(0) and *w_inh_*(0) are the initial synaptic weights for the excitatory and inhibitory synapses respectively, and *C_OU_* is the scaling factor for the Ornstein-Uhlenbeck noise injected in the neurons.

The cortical microcircuit was composed of 4000 neurons connected randomly with connection probabilities described in Details to computer simulation 1. The initial synaptic weights of the synapses and the levels of OU noise were tuned to achieve a spontaneous firing rate of about 4.6 Hz, while maintaining an asynchronous irregular firing activity in the circuit. 50% of all neurons (randomly chosen, 50% excitatory and 50% inhibitory) received downscaled OU noise (by a factor 0.2 from the model reported in [26]), with the subtracted part substituted by additional synaptic input from the circuit. The input connection probabilities of these neurons were scaled up, so that the firing rates remain in the same range as for the other neurons. This was done in order to observe how the learning mechanisms work when most of the input conductance in the neuron comes from a larger number of input synapses which are plastic, rather than from a static noise process. The reinforced neurons were randomly chosen from this group of neurons.

We chose a smaller microcircuit, composed of 540 neurons, for the computer simulation 5 in order to be able to perform a large number of training trials. The synaptic weights in this smaller circuit were chosen (see Table 3) to achieve an appropriate level of firing activity in the circuit that is modulated by the external input. The circuit neurons had injected an Ornstein-Uhlenbeck (OU) noise multiplied by 0.4 in order to emulate the background synaptic activity in neocortical neurons in vivo, and test the learning in a more biologically realistic settings. This produced significant trial-to-trial variability in the circuit response (see Figure 10D). A lower value of the noise level could also be used without affecting the learning, whereas increasing the amount of injected noise would slowly deteriorate the information that the circuit activity maintains about the injected inputs, resulting in a decline of the learning performance.

### Readout Neurons

The maximum values of the synaptic weights of readout neurons for computer simulations 2, 4, and 5, together with the number of synapses of the neurons, are given in Table 4.

**Table 4:** Specific parameter values for the trained (readout) neurons in computer simulation 2, 4, and 5.

Simulation No.	Num. Synapses	*w_max_* [nS]	*C_OU_*
2	100	11.9	1.0
4	200	5.73	0.2
5	432	2.02	0.2

*w_max_* is the upper hard bound of the synaptic weights of the synapses. *C_OU_* is the scaling factor for the Ornstein-Uhlenbeck noise injected in the neurons.

The neuron in computer simulation 2 had 100 synapses. We chose 200 synapses for the neuron in computer simulation 4, in order to improve the learning performance. Such improvement of the learning performance for larger numbers of synapses is in accordance with our theoretical analysis (see Equation 17), since for learning the classification of temporal patterns the temporal variation of the voltage of the postsynaptic membrane turns out to be of critical importance (see the discussion after Equation 17). This temporal variation depends less on the shape of a single EPSP and more on the temporal pattern of presynaptic firing when the number of synapses is increased. In computer simulation 5 the readout neuron received inputs from all 432 excitatory neurons in the circuit. The synaptic weights were chosen in accordance with the number of synapses in order to achieve a firing rate suitable for the particular task, and to balance the synaptic input and the noise injections in the neurons.

For the pattern discrimination task (computer simulation 4) and the speech recognition task (computer simulation 5), the amount of noise had to be chosen to be high enough to achieve sufficient variation of the membrane potential from trial to trial near the firing threshold, and low enough so that it would not dominate the fluctuations of the membrane potential. In the experiment where the exact spike times were rewarded (computer simulation 2), the noise had a different role. As described in the Results section, there the noise effectively controls the amount of depression. If the noise (and therefore the depression) is too weak, w* = 0 synapses do not converge to 0. If the noise is too strong, w* = *w_max_* synapses do not converge to *w_max_*. To achieve the desired learning result, the noise level should be in a range where it reduces the correlations of the synapses with w* = 0 so that the depression of STDP will prevail, but at the same time is not strong enough to do the same for the other group of synapses with w* = *w_max_*, since they have stronger pre-before-post correlations. For our simulations, we have set the noise level to the full amount of OU noise.

### Details to Computer Simulation 1: Model for Biofeedback Experiment

The cortical microcircuit model consisted of 4000 neurons with twenty percent of the neurons randomly chosen to be inhibitory, and the others excitatory. The connections between the neurons were created randomly, with different connectivity probabilities depending on whether the postsynaptic neuron received the full amount of OU noise, or downscaled OU noise with an additional compensatory synaptic input from the circuit. For neurons in the latter sub-population, the connection probabilities were *p_ee_* = 0.02, *p_ei_* = 0.02, *p_ie_* = 0.024 and *p_ii_* = 0.016 where the ee, ei, ie, ii indices designate the type of the presynaptic and postsynaptic neurons (e = excitatory or i = inhibitory). For the other neurons the corresponding connection probabilities were downscaled by 0.4. The resulting firing rates and correlations for both types of excitatory neurons are plotted in Figure S1 and Figure S2.

The shape of the reward kernel *ε_r_*(*t*) was chosen as a difference of two *α*-functions
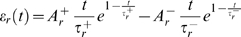
(29)one positive *α*-pulse with a peak at 0.4 sec after the corresponding spike, and one long-tailed negative *α*-pulse which makes sure that the integral over the reward kernel is zero. The parameters for the reward kernel were 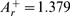, 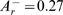, 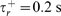, 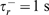, and *d_r_* = 0.2 s, which produced a peak value of the reward pulse 0.4 s after the spike that caused it.

### Details to Computer Simulation 2: Learning Spike Times

We used the following function for the reward kernel *κ*(*r*)
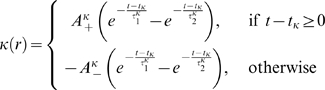
(30)where 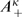 and 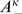 are positive scaling constants, 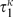 and 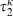 define the shape of the two double-exponential functions the kernel is composed of, and *t_κ_* defines the offset of the zero-crossing from the origin. The parameter values used in our simulations were 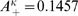, 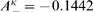, 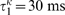, 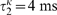 and *t_κ_* = −1 ms. The reward delay was equal to *d_r_* = 0.4 s.

### Details to Computer Simulation 3: Testing the Analytically Derived Conditions

We used a linear Poisson neuron model as in the theoretical analysis with static synapses and exponentially decaying postsynaptic responses 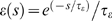. The neuron had 100 excitatory synapses, except in experiment #6, where we used 200 synapses. In all experiments the target neuron received additional 10 excitatory synapses with weights set to *w_max_*. The input spike trains were Poisson processes with a constant rate of *r_pre_* = 6 Hz, except in experiment # 6 where the rate was *r_pre_* = 3 Hz. The weights of the target neuron were set to 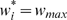 for 0≤*i*<50 and 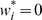 for 50≤*i*<100.

The time constants of the reward kernel were 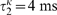, whereas 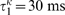 had different values in different experiments (reported in table 1). The value of *t_κ_* was always set to an optimal value such that the 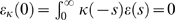. The time constant *τ*_−_ of the negative part of the STDP window function *W*(*r*) was set to *τ*_+_. The reward signal was delayed by *τ_d_* = 0.4 s. The simulations were performed for varying durations of simulated biological time (see the *t_sim_*-column in Table 1).

### Details to Computer Simulation 4: Learning Pattern Classification

We used the reward signal from Equation 16, with an *α*-function for the reward kernel 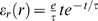, and the reward delay *d_r_* set to 300 ms. The amplitudes of the positive and negative pulses were *α_P_* = −*α_N_* = 1.435 and the time constant of the reward kernel was *τ* = 100 ms.

### Details to Computer Simulation 5: Training a Readout Neuron with Reward-Modulated STDP To Recognize Isolated Spoken Digits

#### Spike representations of speech utterances

The speech utterances were preprocessed by the cochlea model described in [43], which captures the filtering properties of the cochlea and hair cells in the human inner ear. The resulting analog signals were encoded by spikes with the BSA spike encoding algorithm described in [44]. We used the same preprocessing to generate the spikes as in [45]. The spike representations had a duration of about 400 ms and 20 input channels. The input channels were connected topographically to the cortical microcircuit model. The neurons in the circuit were split into 20 disjunct subsets of 27 neurons, and each input channel was connected to the 27 neurons in its corresponding subsets. The readout neuron was trained with 20 different spike inputs to the circuit, where 10 of them resulted from utterances of digit “one”, and the other 10 resulted from utterances of digit “two” by the same speaker.

#### Training procedure

We performed 2000 training trials, where for each trial a spike representation of a randomly chosen utterance out of 10 utterances for one digit was injected into the circuit. The digit changed from trial to trial. Whenever the readout neuron spiked during the presentation of an utterance of digit “two”, a positive pulse was generated in the reward signal, and accordingly, for utterances of digit “one”, a negative pulse in the reward was generated. We used the reward signal from Equation 16. The amplitudes of the positive and negative pulses were *α_P_* = −*α_N_* = 0.883. The time constant of the reward kernel *ε_r_*(*r*) was *τ* = 100 ms. The pulses in the reward were delayed *d_r_* = 300 ms from the spikes that caused them.

#### Cortical microcircuit details

The cortical microcircuit model consisted of 540 neurons with twenty percent of the neurons randomly chosen to be inhibitory, and the others excitatory. The recurrent connections in the circuit were created randomly with a connection probability of 0.1. Long-term plasticity was not modeled in the circuit synapses.

The synapses for the connections from the input neurons to the circuit neurons were static, current based with axon conduction delay of 1 ms, and exponentially decaying PSR with time constant *τ_e_* = 3 ms and amplitude *w_input_* = 0.715 nA.

## Discussion

We have presented in this article analytical tools which make it possible to predict under which conditions reward-modulated STDP will achieve a given learning goal in a network of neurons. These conditions specify relationships between parameters and auxiliary functions (learning curves for STDP, eligibility traces, reward signals etc.) that are involved in the specification of the reward-modulated STDP learning rule. Although our analytical results are based on some simplifying assumptions, we have shown that they predict quite well the outcomes of computer simulations of quite complex models for cortical networks of neurons.

We have applied this learning theory for reward-modulated STDP to a number of biologically relevant learning tasks. We have shown that the biofeedback result of Fetz and Baker [17] can in principle be explained on the basis of reward-modulated STDP. The underlying credit assignment problem was extremely difficult, since the monkey brain had no direct information about the identity of the neuron whose firing rate was relevant for receiving rewards. This credit assignment problem is even more difficult from the perspective of a single synapse, and hence for the application of a local synaptic plasticity rule such as reward-modulated STDP. However our theoretical analysis (see Equations 10 and 11) has shown that the longterm evolution of synaptic weights depended only on the correlation of pairs of pre- and postsynaptic spikes with the reward signal. Therefore the firing rate of the rewarded neuron increased (for a computer simulation of a recurrent network consisting of 4000 conductance based LIF neurons with realistic background noise typical for in-vivo conditions, and 228954 synapses that exhibited data-based short term synaptic plasticity) within a few minutes of simulated biological time, like in the experimental data of [17], whereas the firing rates of the other neurons remained invariant (see Figure 4B). We were also able to model differential reinforcement of two neurons in this way (Figure 2). These computer simulations demonstrated a remarkable stability of the network dynamics (see Figures 2A, 4A, and 5) in spite of the fact that all excitatory synapses were continuously subjected to reward-modulated STDP. In particular, the circuit remained in the asynchronous irregular firing regime, that resembles spontaneous firing activity in the cortex [9]. Other STDP-rules (without reward modulation) that maintain this firing regime have previously been exhibited in [22]. It was also reported in [17], and further examined in [46], that bursts of the reinforced neurons were often accompanied by activations of specific muscles in the biofeedback experiment by Fetz and Baker. But the relationship between bursts of the recorded neurons in precentral motor cortex and muscle activations was reported to be quite complex and often dropped out after continued reinforcement of the neuron alone. Furthermore in [46] it was shown that all neurons tested in that study could be dissociated from their correlated muscle activity by differentially reinforcing simultaneous suppression of EMG activity. These results suggest that the solution of the credit assignment problem by the monkeys (to stronger activate that neuron out of billions of neurons in their precentral gyrus that was reinforced) may have been supported by large scale exploration strategies that were associated with muscle activations. But the previously mentioned results on differential reinforcements of two nearby neurons suggest that this large scale exploration strategy had to be complemented by exploration on a finer spatial scale that is difficult to explain on the basis of muscle activations (see [19] for a detailed discussion).

Whereas this learning task focused on firing rates, we have also shown (see Figure 7) that neurons can learn via reward-modulated STDP to respond to inputs with particular spike trains, i.e., particular temporal output patterns. It has been pointed out in [27] that this is a particularly difficult learning task for reward-modulated STDP, and it was shown there that it can be accomplished with a modified STDP rule and more complex reward prediction signals without delays. We have complemented the results of [27] by deriving specific conditions (Equations 13–15) under which this learning task can be solved by the standard version of reward-modulated STDP. Extensive computer simulations have shown that these analytically derived conditions for a simpler neuron model predict also for a LIF neuron with conductance based synapses whether it is able to solve this learning task. Figure 8 shows that this learning theory for reward-modulated STDP is also able to predict quite well *how fast* a neuron can learn to produce a desired temporal output pattern. An interesting aspect of [27] is that there also the utility of third signals that provide information about changes in the expectation of reward was explored. We have considered in this article only learning scenarios where reward prediction is not possible. A logical next step will be to extend our learning theory for reward-modulated STDP to scenarios from classical reinforcement learning theory that include reward prediction.

We have also addressed the question to what extent neurons can learn via reward-modulated STDP to respond with different firing rates to different spatio-temporal presynaptic firing patterns. It had already been shown in [12] that this learning rule enables neurons to classify spatial firing patterns. We have complemented this work by deriving an analytic expression for the expected weight change in this learning scenario (see Equation 17), which clarifies to what extent a neuron can learn by reward-modulated STDP to distinguish differences in the temporal structure of presynaptic firing patterns. This theoretical analysis showed that in the extreme case, where all incoming information is encoded in the relative timing of presynaptic spikes, reward-modulated STDP is not able to produce a higher average membrane potential for selected presynaptic firing patterns, even if that would be rewarded. But it is able to increase the variance of the membrane potential, and thereby also the number of spikes of any neuron model that has (unlike the simple linear Poisson neuron) a firing threshold. The simulation results in Figure 9 confirm that in this way a LIF neuron can learn with the standard version of reward-modulated STDP to discriminate even purely temporal presynaptic firing patterns, by producing more spikes in response to one of these patterns.

A surprising feature is, that although the neuron was rewarded here only for responding with a higher firing rate to one presynaptic firing pattern *P*, it automatically started to respond to this pattern *P* with a specific temporal spike pattern, that advanced in time during training (see Figure 9A).

Finally, we have shown that a spiking neuron can be trained by reward-modulated STDP to read out information from a simulated cortical microcircuit (see Figure 10). This is insofar of interest, as previous work [31],[34],[47] had shown that models of generic cortical microcircuits have inherent capabilities to serve as preprocessors for such readout neurons, by combining in diverse linear and nonlinear ways information that was contained in different time segments of spike inputs to the circuit (“liquid computing model”). The classification of spoken words (that were first transformed into spike trains) had been introduced as a common benchmark task for the evaluation of different approaches towards computing with spiking neurons [31]–[33],[45],[48]. But so far all approaches that were based on learning (rather than on clever constructions) had to rely on supervised training of a simple linear readout. This gave rise to the question whether also biologically more realistic models for readout neurons can be trained through a biologically more plausible learning scenario to classify spoken words. The results of Figure 10 may be interpreted as a tentative positive answer to this question. We have demonstrated that LIF neurons with conductance based synapses (that are subject to biologically realistic short term plasticity) can learn without a supervisor through reward-modulated STDP to classify spoken digits. In contrast to the result of Figure 9, the output code that emerged here was a rate code. This can be explained through the significant in-class variance of circuit responses to different utterances of the same word (see Figure 10C and 10D). Although the LIF neuron learnt here without a supervisor to respond with different firing rates to utterances of different words by the same speaker (whereas the rate output was very similar for both words at the beginning of learning, see Figure 10E), the classification capability of these neurons has not yet reached the level of linear readouts that are trained by a supervisor (for example, speaker independent word classification could not yet be achieved in this way). Further work is needed to test whether the classification capability of LIF readout neurons can be improved through additional preprocessing in the cortical microcircuit model, through a suitable variation of the reward-modulated STDP rule, or through a different learning scenario (mimicking for example preceding developmental learning that also modifies the presynaptic circuit).

The new learning theory for reward-modulated STDP will also be useful for biological experiments that aim at the clarification of details of the biological implementation of synaptic plasticity in different parts of the brain, since it allows to make predictions which types and time courses of signals would be optimal for a particular range of learning tasks. For each of the previously discussed learning tasks, the theoretical analysis provided conditions on the structure of the reward signal *d*(*t*) which guaranteed successful learning. For example, in the biofeedback learning scenario (Figure 4), every action potential of the reinforced neuron led—after some delay—to a change of the reward signal *d*(*t*). The shape of this change was defined by the reward kernel *ε*(*r*). Our analysis revealed that this reward kernel can be chosen rather arbitrarily as long as the integral over the kernel is zero, and the integral over the product of the kernel and the eligibility function is positive. For another learning scenario, where the goal was that the output spike train 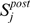 of some neuron *j* approximates the spike timings of some target spike train S* (Figure 7), the reward signal has to depend on both, 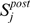 and S*. The dependence of the reward signal on these spike timings was defined by a reward kernel *κ*(*r*). Our analysis showed that the reward kernel has to be chosen for this task so that the synapses receive positive rewards if the postsynaptic neuron fires close to the time of a spike in the target spike train S* or somewhat later, and negative rewards when an output spike occurs in the order of ten milliseconds too early. In the pattern discrimination task of Figure 9 each postsynaptic action potential was followed—after some delay—by a change of the reward signal which depended on the pattern presented. Our theoretical analysis predicted that this learning task can be solved if the integrals 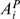 and 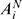 defined by Equation 18 are such that 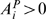 and 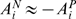. Again, this constraints are fulfilled for a large class of reward kernels, and a natural choice is to use a non-negative reward kernel *ε_r_*. There are currently no data available on the shape of reward kernels in biological neural systems. The previous sketched theoretical analysis makes specific prediction for the shape of reward kernels (depending on the type of learning task in which a biological neural system is involved) which can potentially be tested through biological experiments.

An interesting general aspect of the learning theory that we have presented in this article is that it requires substantial trial-to-trial variability in the neural circuit, which is often viewed as “noise” of imperfect biological implementations of theoretically ideal circuits of neurons. This learning theory for reward-modulated STDP suggests that the main functional role of noise is to maintain a suitable level of spontaneous firing (since if a neuron does not fire, it cannot find out whether this will be rewarded), which should vary from trial to trial in order to explore which firing patterns are rewarded (It had been shown in [31],[34],[47] that such highly variable circuit activity is compatible with a stable performance of linear readouts). On the other hand if a neuron fires primarily on the basis of a noise current that is directly injected into that neuron, and not on the basis of presynaptic activity, then STDP does not have the required effect on the synaptic connections to this neuron (see Figure S6). This perspective opens the door for subsequent studies that compare for concrete biological learning tasks the theoretically derived optimal amount and distribution of trial-to-trial variability with corresponding experimental data.

### Related Work

The theoretical analysis of this model is directly applicable to the learning rule considered in [12]. There, the network behavior of reward-modulated STDP was also studied some situations different from the ones in this article. The computer simulations of [12] operate apparently in a different dynamic regime, where LTD dominates LTP in the STDP-rule, and most weights (except those that are actively increased through reward-modulated STDP) have values close to 0 (see Figure 1b and 1d in [12], and compare with Figure 5 in this article). This setup is likely to require for successful learning a larger dominance of pre-before-post over post-before-pre pairs than the one shown in Figure 4E. Furthermore, whereas a very low spontaneous firing rate of 1 Hz was required in [12], computer simulation 1 shows that reinforcement learning is also feasible at spontaneous firing rates which correspond to those reported in [17] (the preceding theoretical analysis had already suggested that the success of the model does not depend on particularly low firing rates). The articles [15] and [13] investigate variations of reward-modulated STDP rules that do not employ learning curves for STDP that are based on experimental data, but modified curves that arise in the context of a very interesting top-down theoretical approach (distributed reinforcement learning [14]). The authors of [16] arrive at similar learning rules in a supervised scenario which can be reinterpreted in the context of reinforcement learning. We expect that a similar theory as we have presented in this article for the more commonly discussed version of STDP can also be applied to their modified STDP rules, thereby making it possible to predict under which conditions their learning rules will succeed. Another reward based learning rule for spiking neurons was recently presented in [49]. This rule exploits correlations of a reward signal with noisy perturbations of the neuronal membrane conductance in order to optimize some objective function. One crucial assumption of this approach is that the synaptic plasticity mechanism “knows” which contributions to the membrane potential arise from synaptic inputs, and which contributions are due to internal noise. Such explicit knowledge of the noise signal is not needed in the reward-modulated STDP rule of [12], which we have considered in this article. The price one has to pay for this potential gain in biological realism is a reduced generality of the learning capabilities. While the learning rule in [49] approximates gradient ascent on the objective function, this cannot be stated for reward-modulated STDP at present. Timing-based pattern discrimination with a spiking neuron, as discussed in the section “Pattern discrimination with reward-modulated STDP” of this article, was recently tackled in [50]. The authors proposed the tempotron learning rule, which increases the peak membrane voltage for one class of input patterns (if no spike occurred in response to the input pattern) while decreasing the peak membrane voltage for another class of input patterns (if a spike occurred in response to the pattern). The main difference between this learning rule and reward-modulated STDP is that the tempotron learning rule is sensitive to the peak membrane voltage, whereas reward-modulated STDP is sensitive to local fluctuations of the membrane voltage. Since the time of the maximal membrane voltage has to be determined for each pattern by the synaptic plasticity mechanism, the basic tempotron rule is perhaps not biologically realistic. Therefore, an approximate and potentially biologically more realistic learning rule was proposed in [50], where plasticity following error trials is induced at synapse *i* only if the voltage within the postsynaptic integration time after their activation exceeds a plasticity threshold *κ*. One potential problem of this rule is the plasticity threshold *κ*, since a good choice of this parameter strongly depends on the mean membrane voltage after input spikes. This problem is circumvented by reward-modulated STDP, which considers instead the local change in the membrane voltage. Further work is needed to compare the advantages and disadvantages of these different approaches.

### Conclusion

Reward-modulated STDP is a very promising candidate for a synaptic plasticity rule that is able to orchestrate local synaptic modifications in such a way that particular functional properties of larger networks of neurons can be achieved and maintained (we refer to [12] and [27] for discussion of potential biological implementations of this plasticity rule). We have provided in this article analytical tools which make it possible to evaluate this rule and variations of this rule not just through computer simulations, but through theoretical analysis. In particular we have shown that successful learning is only possible if certain relationships hold between the parameters that are involved. Some of these predicted relationships can be tested through biological experiments.

Provided that these relationships are satisfied, reward-modulated STDP turns out to be a powerful rule that can achieve self-organization of synaptic weights in large recurrent networks of neurons. In particular, it enables us to explain seemingly inexplicable experimental data on biofeedback in monkeys. In addition reward-modulated STDP enables neurons to distinguish complex firing patterns of presynaptic neurons, even for data-based standard forms of STDP, and without the need for a supervisor that tells the neuron when it should spike. Furthermore reward-modulated STDP requires substantial spontaneous activity and trial-to-trial variability in order to support successful learning, thereby providing a functional explanation for these ubiquitous features of cortical networks of neurons. In fact, not only spontaneous activity but also STDP itself may be seen in this context as a mechanism that supports the exploration of different firing chains within a recurrent network, until a solution is found that is rewarded because it supports a successful computational function of the network.

## Supporting Information

Figure S1 Variations of Figure 5B–D for those excitatory neurons which receive the full amount of Ornstein-Uhlenbeck noise. (B) The distribution of the firing rates of these neurons remains unchanged during the simulation. The colors of the curves and the corresponding intervals are as follows: red (300–360 sec), green (600–660 sec), blue (900–960 sec), magenta (1140–1200 sec). (C) Cross-correlogram of the spiking activity of these neurons, averaged over 200 pairs of neurons and over 60 s, with a bin size of 0.2 ms, for the period between 300 and 360 seconds of simulation time. It is calculated as the cross-covariance divided by the square root of the product of variances. (D) As in (C), but for the last 60 seconds of the simulation. The correlation statistics in the circuit is stable during learning.
(0.06 MB PDF)

Figure S2 Variations of Figure 5B–D for those excitatory neurons which receive a reduced amount of Ornstein-Uhlenbeck noise, but receive more synaptic inputs from other neurons. (B) The distribution of the firing rates of these neurons remains unchanged during the simulation. The colors of the curves and the corresponding intervals are as follows: red (300–360 sec), green (600–660 sec), blue (900–960 sec), magenta (1140–1200 sec). (C) Cross-correlogram of the spiking activity in the circuit, averaged over 200 pairs of these neurons and over 60 s, with a bin size of 0.2 ms, for the period between 300 and 360 seconds of simulation time. It is calculated as the cross-covariance divided by the square root of the product of variances. (D) As in (C), but for the last 60 seconds of the simulation. The correlation statistics in the circuit is stable during learning.
(0.06 MB PDF)

Figure S3Variation of Figure 4 from computer simulation 1 with results from a simulation where the weight-dependent version of STDP proposed in [22] was used. This STDP rule is defined by the following equations: 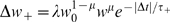 and 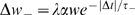. We used the parameters proposed in [36], i.e. *μ* = 0.4, *α* = 0.11, *τ*_+_ = *τ*_−_ = 20 ms, *λ* = 0.1 and *w*_0_ = 272.6 pS. The *w*_0_ parameter was calculated according to the formula: 
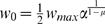
 where *w_max_* is the maximum synaptic weight of the synapse. The amplitude parameters 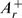, 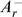 for the reward kernel were set to 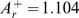 and 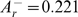. All other parameter values were the same as in computer simulation 1.(0.09 MB PDF)

Figure S4 Variation of Figure 5 for the weight-dependent STDP rule from [22] (as in Figure S3).
(0.06 MB PDF)

Figure S5Variation of Figure 7 (i.e., of computer simulation 2) for a simulation where we used current-based synapses without short-term plasticity. The post-synaptic response had an exponentially decaying form 
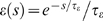
, with *τ_ε_* = 5 ms. The value of the maximum synaptic weight was *w_max_* = 32.9 pA. All other parameter values were the same as in computer simulation 2.(0.17 MB PDF)

Figure S6 Dependence of the learning performance on the noise level in computer simulation 2. The angular error (defined as the angle between the weight vector **w** of the trained neuron at the end of the simulation and the weight vector w* of the neuron μ*) is taken as measure for the learning performance, and plotted for 9 simulations with different noise levels that are given on the X axis (in term of multiples of the noise level chosen for Figure 7). All other parameters values were the same as in computer simulation 2. The figure shows that the learning performance declines both for too little and for too much noise.
(0.02 MB PDF)

Figure S7Variation of Figure 9 (i.e., of computer simulation 4) with the weight-dependent STDP rule proposed in [22]. This rule is defined by the following equations: 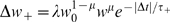 and 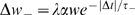. We used the parameters proposed in [22], i.e. *μ* = 0.4, *α* = 0.11, *τ*_+_ = *τ*_−_ = 20 ms, *λ* = 0.1 and *w*_0_ = 72.4 pS. The *w*_0_ parameter was calculated according to the formula: 
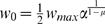
 where *w_max_* is the maximum synaptic weight of the synapse. The amplitude parameters of the reward kernel were set to *α_P_* = −*α_N_* = 1.401. All other parameter values were the same as in computer simulation 4. The variance of the membrane potential increased for pattern *P* from 2.35 (mV)^2^ to 3.66 (mV)^2^ (C), and decreased for pattern *N* (D), from 2.27 (mV)^2^ to 1.54 (mV)^2^.(0.31 MB PDF)

Figure S8Variation of Figure 9 for a simulation where we used current-based synapses without short-term plasticity. The post-synaptic response had an exponentially decaying form 
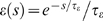
, with *τ_ε_* = 5 ms. The value of the maximum synaptic weight was *w_max_* = 106.2 pA All other parameter values were the same as in computer simulation 4. The variance of the membrane potential increased for pattern *P* from 2.84 (mV)^2^ to 5.89 (mV)^2^ (C), and decreased for pattern *N* (D), from 2.57 (mV)^2^ to 1.22 (mV)^2^.(0.31 MB PDF)

Figure S9Variation of Figure 10 (i.e., of computer simulation 5) for a simulation where we used current-based synapses without short-term plasticity. The post-synaptic response had an exponentially decaying form 
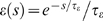
, with *τ_ε_* = 5 ms. The synaptic weights of the excitatory and inhibitory synapses in the cortical microcircuit were set to *w_exc_* = 65.4 pA and *w_inh_* = 238 pA respectively. The maximum synaptic weight of the synapses to the readout neuron was *w_max_* = 54.3 pA. All other parameter values were the same as in computer simulation 5.(0.27 MB PDF)

Figure S10 Spike encodings of 10 utterances of digit “one” by one speaker with the Lyon cochlea model [43], which were used as circuit inputs for computer simulation 5.
(0.05 MB PDF)

Figure S11 Spike encodings of 10 utterances of digit “two” by one speaker with the Lyon cochlea model [43], which were used as circuit inputs for computer simulation 5.
(0.05 MB PDF)
