# Supplementary material for: A Learning Theory for Reward-Modulated Spike-Timing-Dependent Plasticity with Application to Biofeedback
Source: PLoS Comput Biol. 2008 Oct 10;4(10):e1000180. doi: 10.1371/journal.pcbi.1000180 (PMC2543108; doi:10.1371/journal.pcbi.1000180)
Supplement: Figure S1 — Variations of Figure 5B–D for those excitatory neurons which receive the full amount of Ornstein-Uhlenbeck noise. (B) The distribution of the firing rates of these neurons remains unchanged during the simulation. The colors of the curves and the corresponding intervals are as follows: red (300–360 sec), green (600–660 sec), blue (900–960 sec), magenta (1140–1200 sec). (C) Cross-correlogram of the spiking activity of these neurons, averaged over 200 pairs of neurons and over 60 s, with a bin size of 0.2 ms, for the period between 300 and 360 seconds of simulation time. It is calculated as the cross-covariance divided by the square root of the product of variances. (D) As in (C), but for the last 60 seconds of the simulation. The correlation statistics in the circuit is stable during learning. (0.06 MB PDF) [file pcbi.1000180.s001.pdf]

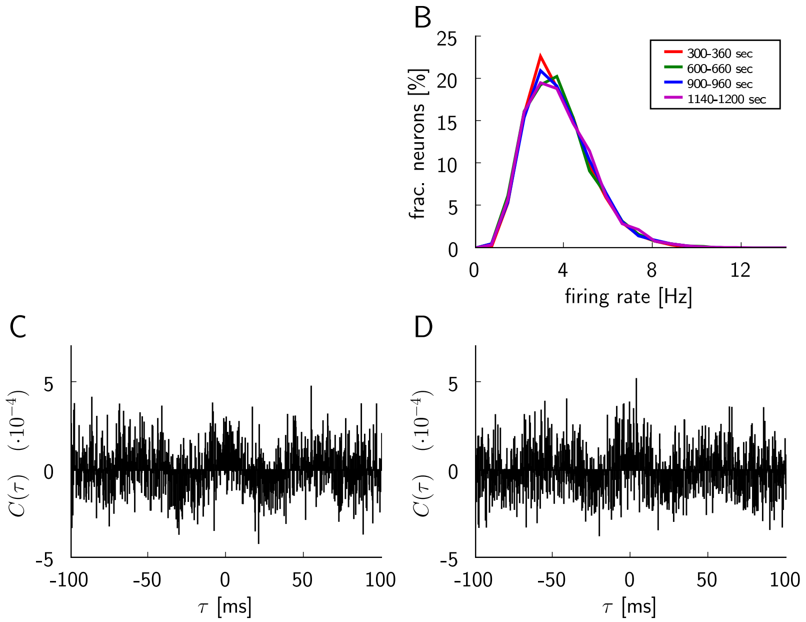

Figure S1: Variations of panels **B) - D)** in Fig. 5 for those excitatory neurons which receive the full amount of Ornstein-Uhlenbeck noise. **B)** The distribution of the firing rates of these neurons remains unchanged during the simulation. The colors of the curves and the corresponding intervals are as follows: red ($300-360$ sec), green ($600-660$ sec), blue ($900-960$ sec), magenta ($1140-1200$ sec). **C)** Cross-correlogram of the spiking activity of these neurons, averaged over 200 pairs of neurons and over 60 s, with a bin size of 0.2 ms, for the period between 300 and 360 seconds of simulation time. It is calculated as the cross-covariance divided by the square root of the product of variances. **D)** As in **C)**, but for the last 60 seconds of the simulation. The correlation statistics in the circuit is stable during learning.
