# Supplementary material for: A Learning Theory for Reward-Modulated Spike-Timing-Dependent Plasticity with Application to Biofeedback
Source: PLoS Comput Biol. 2008 Oct 10;4(10):e1000180. doi: 10.1371/journal.pcbi.1000180 (PMC2543108; doi:10.1371/journal.pcbi.1000180)
Supplement: Figure S3 — Variation of Figure 4 from computer simulation 1 with results from a simulation where the weight-dependent version of STDP proposed in [22] was used. This STDP rule is defined by the following equations: and . We used the parameters proposed in [36], i.e. μ = 0.4, α = 0.11, τ + = τ − = 20 ms, λ = 0.1 and w 0 = 272.6 pS. The w 0 parameter was calculated according to the formula: where wmax is the maximum synaptic weight of the synapse. The amplitude parameters , for the reward kernel were set to and . All other parameter values were the same as in computer simulation 1. (0.09 MB PDF) [file pcbi.1000180.s003.pdf]

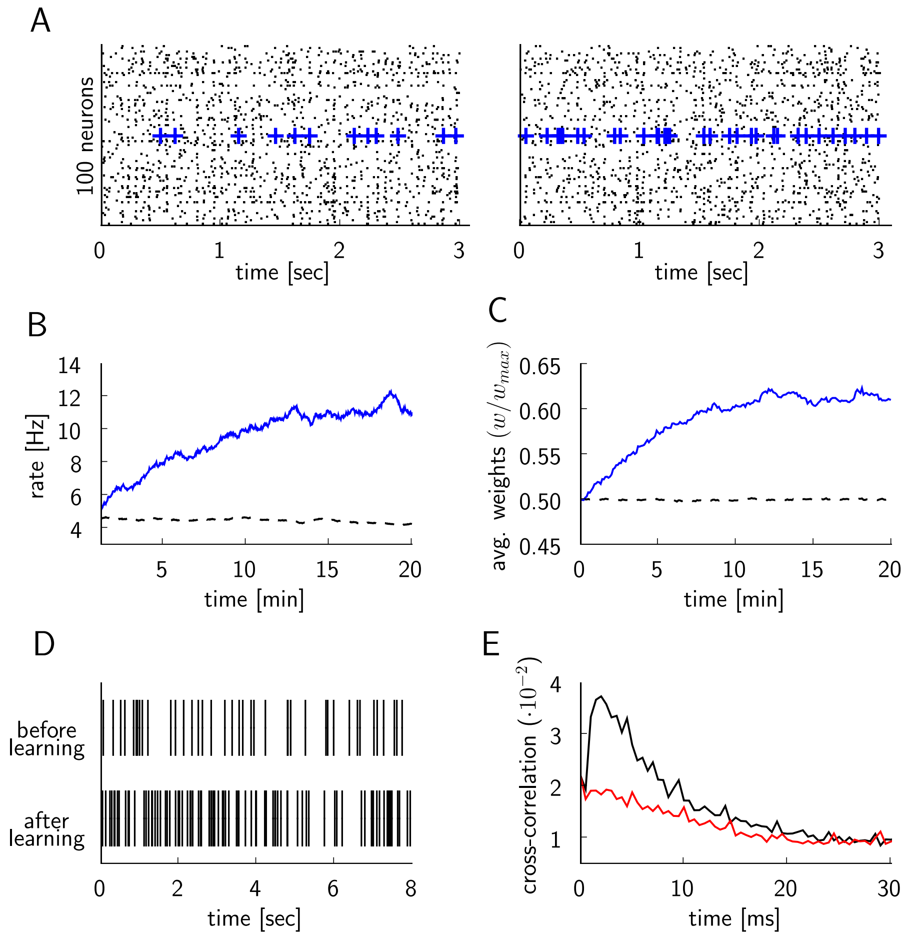

Figure S3: Variation of Fig. 3 from computer simulation 1 with results from a simulation where the weight-dependent version of STDP proposed in [23] was used. This STDP rule is defined by the following equations: $\Delta w_+ = \lambda w_0^{1-\mu} w^\mu e^{-|\Delta t|/\tau_+}$ and $\Delta w_- = \lambda \alpha w e^{-|\Delta t|/\tau_-}$. We used the parameters proposed in [23], i.e. $\mu = 0.4$, $\alpha = 0.11$, $\tau_+ = \tau_- = 20$ms, $\lambda = 0.1$ and $w_0 = 272.6$ pS. The w_0 parameter was calculated according to the formula: $w_0 = \frac{1}{2} w_{max} \alpha^{\frac{1}{1-\mu}}$ where w_{max} is the maximum synaptic weight of the synapse. The amplitude parameters A_r^+, A_r^- for the reward kernel were set to $A_r^+ = 1.104$ and $A_r^- = 0.221$. All other parameter values were the same as in computer simulation 1.
