# Supplementary figures and images for: A Learning Theory for Reward-Modulated Spike-Timing-Dependent Plasticity with Application to Biofeedback
Source: PLoS Comput Biol. 2008 Oct 10;4(10):e1000180. doi: 10.1371/journal.pcbi.1000180 (PMC2543108; doi:10.1371/journal.pcbi.1000180)

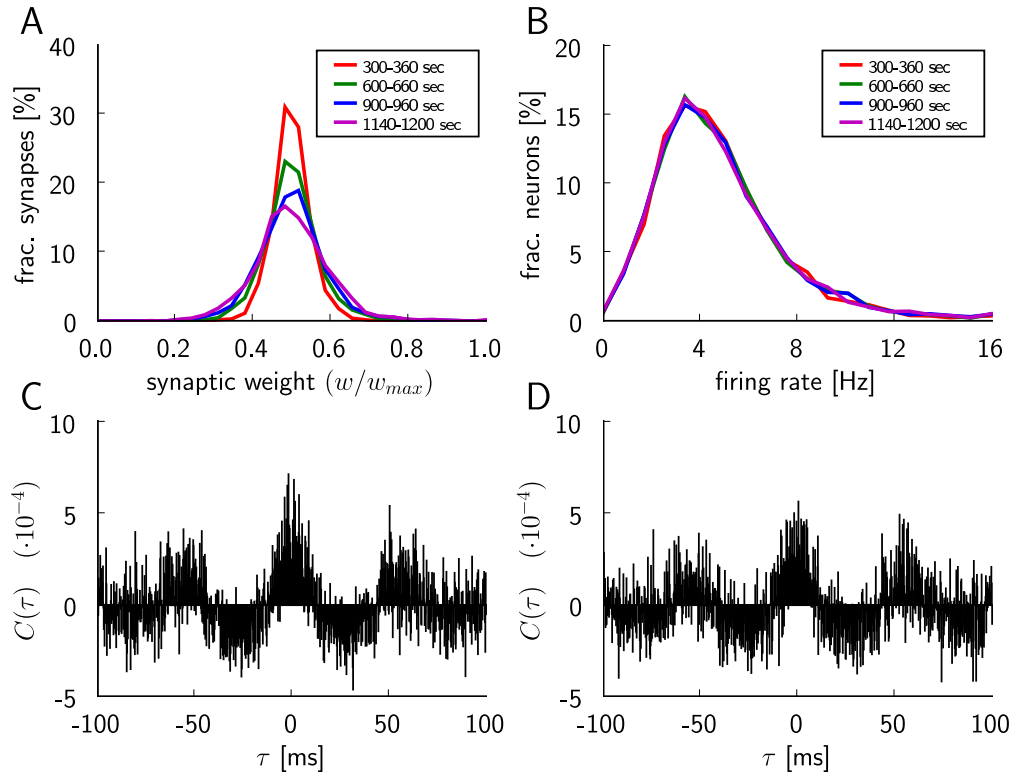

Figure S4: Variation of Fig. 5 for the weight-dependent STDP rule from [23] (as in Fig. S3).

Supplement: Figure S4 — Variation of Figure 5 for the weight-dependent STDP rule from [22] (as in Figure S3). (0.06 MB PDF) [file pcbi.1000180.s004.pdf]
