# Supplementary material for: A Learning Theory for Reward-Modulated Spike-Timing-Dependent Plasticity with Application to Biofeedback
Source: PLoS Comput Biol. 2008 Oct 10;4(10):e1000180. doi: 10.1371/journal.pcbi.1000180 (PMC2543108; doi:10.1371/journal.pcbi.1000180)
Supplement: Figure S5 — Variation of Figure 7 (i.e., of computer simulation 2) for a simulation where we used current-based synapses without short-term plasticity. The post-synaptic response had an exponentially decaying form , with τε = 5 ms. The value of the maximum synaptic weight was wmax = 32.9 pA. All other parameter values were the same as in computer simulation 2. (0.17 MB PDF) [file pcbi.1000180.s005.pdf]

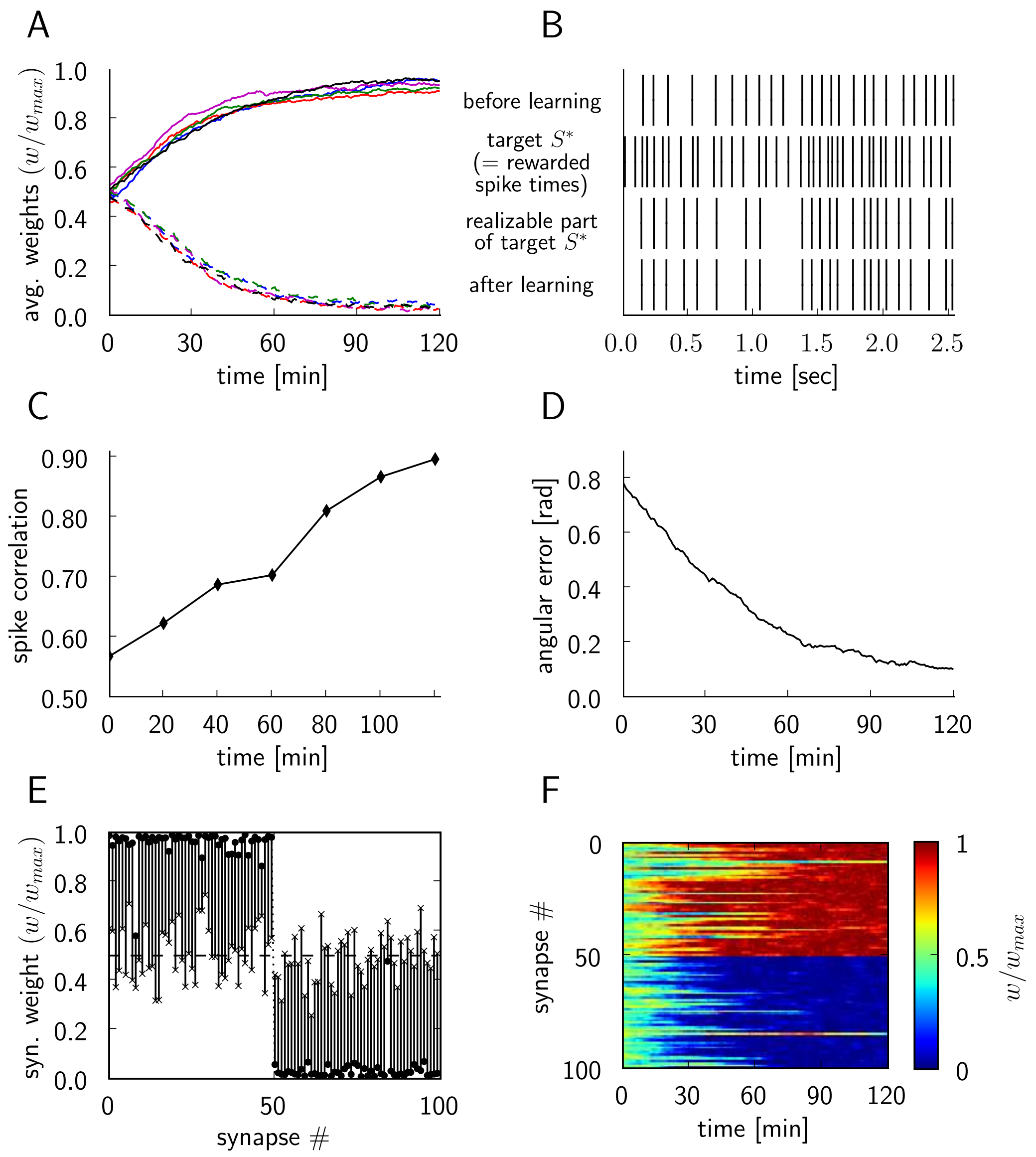


Figure S5: Variation of Fig. 7 (i.e., of computer simulation 2) for a simulation where we used current-based synapses without short-term plasticity. The post-synaptic response had an exponentially decaying form $\epsilon(s) = e^{-s/\tau_\epsilon}/\tau_\epsilon$, with $\tau_\epsilon = 5ms$. The value of the maximum synaptic weight was $w_{max} = 32.9$ pA. All other parameter values were the same as in computer simulation 2.
