# Supplementary material for: A Learning Theory for Reward-Modulated Spike-Timing-Dependent Plasticity with Application to Biofeedback
Source: PLoS Comput Biol. 2008 Oct 10;4(10):e1000180. doi: 10.1371/journal.pcbi.1000180 (PMC2543108; doi:10.1371/journal.pcbi.1000180)
Supplement: Figure S6 — Dependence of the learning performance on the noise level in computer simulation 2. The angular error (defined as the angle between the weight vector w of the trained neuron at the end of the simulation and the weight vector w* of the neuron μ*) is taken as measure for the learning performance, and plotted for 9 simulations with different noise levels that are given on the X axis (in term of multiples of the noise level chosen for Figure 7). All other parameters values were the same as in computer simulation 2. The figure shows that the learning performance declines both for too little and for too much noise. (0.02 MB PDF) [file pcbi.1000180.s006.pdf]

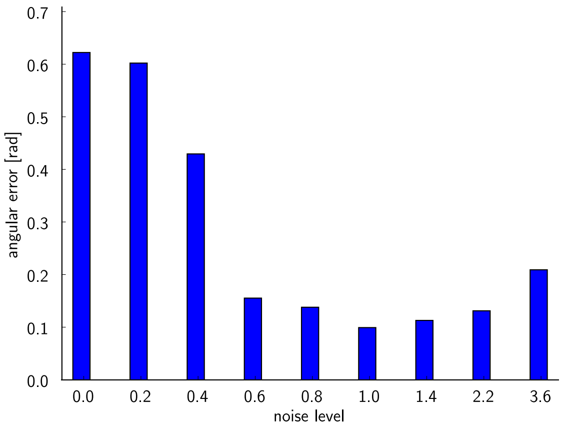

Figure S6: Dependence of the learning performance on the noise level in computer simulation 2. The angular error (defined as the angle between the weight vector $\mathbf{w}$ of the trained neuron at the end of the simulation and the weight vector $\mathbf{w}^*$ of the neuron μ^*) is taken as measure for the learning performance, and plotted for 9 simulations with different noise levels that are given on the X axis (in term of multiples of the noise level chosen for Fig. 7). All other parameters values were the same as in computer simulation 2. The figure shows that the learning performance declines both for too little and for too much noise.
