# Supplementary material for: A Learning Theory for Reward-Modulated Spike-Timing-Dependent Plasticity with Application to Biofeedback
Source: PLoS Comput Biol. 2008 Oct 10;4(10):e1000180. doi: 10.1371/journal.pcbi.1000180 (PMC2543108; doi:10.1371/journal.pcbi.1000180)
Supplement: Figure S7 — Variation of Figure 9 (i.e., of computer simulation 4) with the weight-dependent STDP rule proposed in [22]. This rule is defined by the following equations: and . We used the parameters proposed in [22], i.e. μ = 0.4, α = 0.11, τ + = τ − = 20 ms, λ = 0.1 and w 0 = 72.4 pS. The w 0 parameter was calculated according to the formula: where wmax is the maximum synaptic weight of the synapse. The amplitude parameters of the reward kernel were set to αP = −αN = 1.401. All other parameter values were the same as in computer simulation 4. The variance of the membrane potential increased for pattern P from 2.35 (mV)2 to 3.66 (mV)2 (C), and decreased for pattern N (D), from 2.27 (mV)2 to 1.54 (mV)2. (0.31 MB PDF) [file pcbi.1000180.s007.pdf]

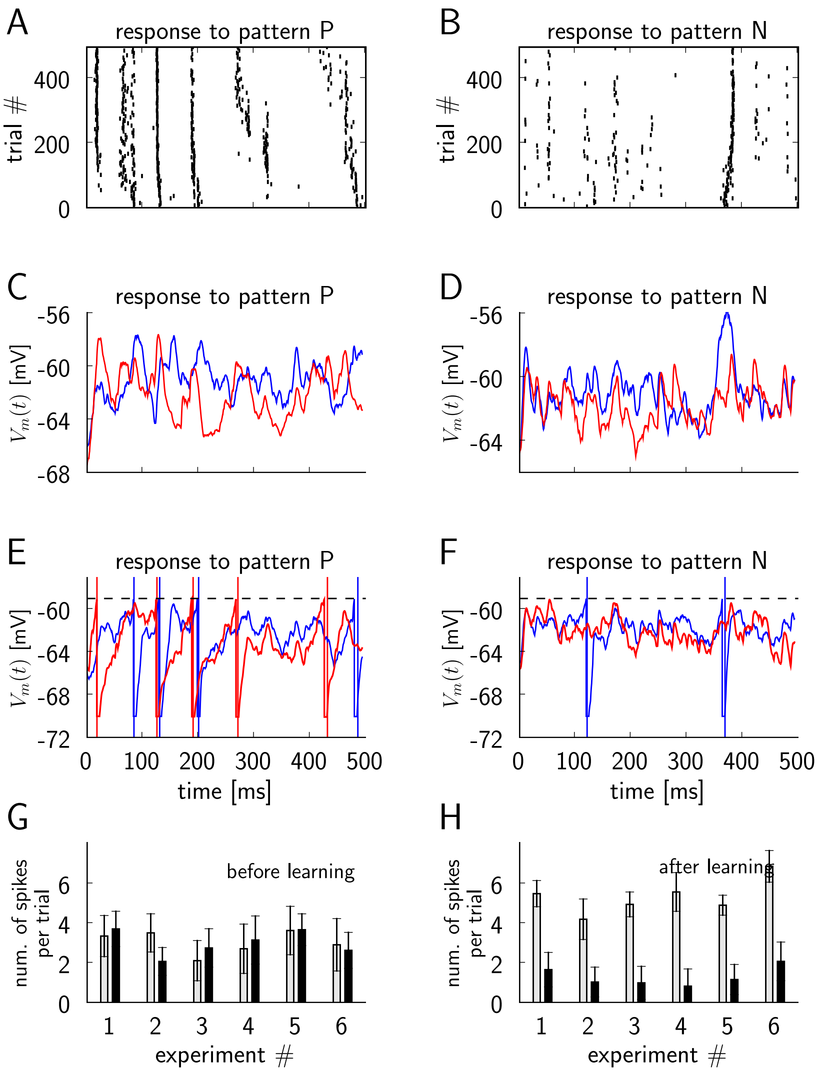

Figure S7: Variation of Fig. 9 (i.e., of computer simulation 4) with the weight-dependent STDP rule proposed in [23]. This rule is defined by the following equations: $\Delta w_+ = \lambda w_0^{1-\mu} w^\mu e^{-|\Delta t|/\tau_+}$ and $\Delta w_- = \lambda \alpha w e^{-|\Delta t|/\tau_-}$. We used the parameters proposed in [23], i.e. $\mu = 0.4$, $\alpha = 0.11$, $\tau_+ = \tau_- = 20\text{ms}$, $\lambda = 0.1$ and $w_0 = 72.4\text{pS}$. The w_0 parameter was calculated according to the formula: $w_0 = \frac{1}{2} w_{max} \alpha^{\frac{1}{1-\mu}}$ where w_{max} is the maximum synaptic weight of the synapse. The amplitude parameters of the reward kernel were set to $\alpha_P = -\alpha_N = 1.401$. All other parameter values were the same as in computer simulation 4. The variance of the membrane potential increased for pattern P from $2.35(mV)^2$ to $3.66(mV)^2$ (panel C), and decreased for pattern N (panel D), from $2.27(mV)^2$ to $1.54(mV)^2$.
