# Supplementary material for: A Learning Theory for Reward-Modulated Spike-Timing-Dependent Plasticity with Application to Biofeedback
Source: PLoS Comput Biol. 2008 Oct 10;4(10):e1000180. doi: 10.1371/journal.pcbi.1000180 (PMC2543108; doi:10.1371/journal.pcbi.1000180)
Supplement: Figure S8 — Variation of Figure 9 for a simulation where we used current-based synapses without short-term plasticity. The post-synaptic response had an exponentially decaying form , with τε = 5 ms. The value of the maximum synaptic weight was wmax = 106.2 pA All other parameter values were the same as in computer simulation 4. The variance of the membrane potential increased for pattern P from 2.84 (mV)2 to 5.89 (mV)2 (C), and decreased for pattern N (D), from 2.57 (mV)2 to 1.22 (mV)2. (0.31 MB PDF) [file pcbi.1000180.s008.pdf]

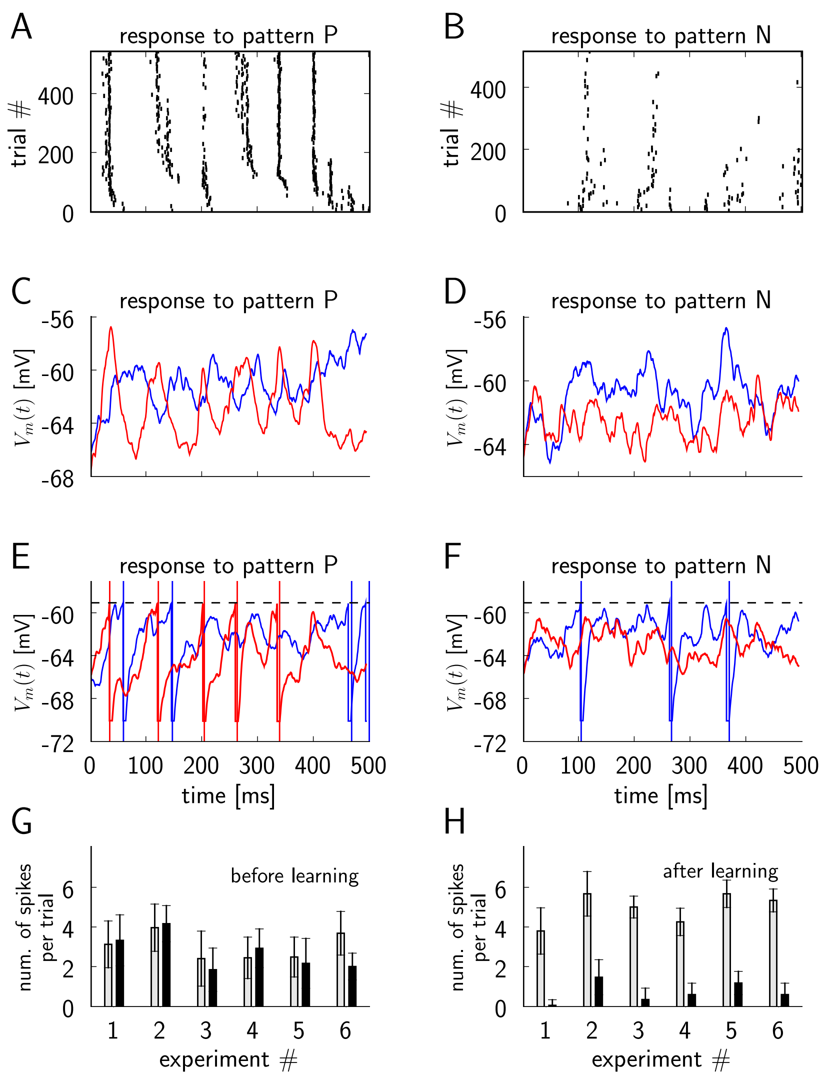

Figure S8: Variation of Fig. 9 for a simulation where we used current-based synapses without short-term plasticity. The post-synaptic response had an exponentially decaying form $\epsilon(s) = e^{-s/\tau_\epsilon}/\tau_\epsilon$, with $\tau_\epsilon = 5ms$. The value of the maximum synaptic weight was $w_{max} = 106.2$ pA All other parameter values were the same as in computer simulation 4. The variance of the membrane potential increased for pattern P from $2.84(mV)^2$ to $5.89(mV)^2$ (panel C), and decreased for pattern N (panel D), from $2.57(mV)^2$ to $1.22(mV)^2$.
