# Supplementary material for: A Learning Theory for Reward-Modulated Spike-Timing-Dependent Plasticity with Application to Biofeedback
Source: PLoS Comput Biol. 2008 Oct 10;4(10):e1000180. doi: 10.1371/journal.pcbi.1000180 (PMC2543108; doi:10.1371/journal.pcbi.1000180)
Supplement: Figure S9 — Variation of Figure 10 (i.e., of computer simulation 5) for a simulation where we used current-based synapses without short-term plasticity. The post-synaptic response had an exponentially decaying form , with τε = 5 ms. The synaptic weights of the excitatory and inhibitory synapses in the cortical microcircuit were set to wexc = 65.4 pA and winh = 238 pA respectively. The maximum synaptic weight of the synapses to the readout neuron was wmax = 54.3 pA. All other parameter values were the same as in computer simulation 5. (0.27 MB PDF) [file pcbi.1000180.s009.pdf]

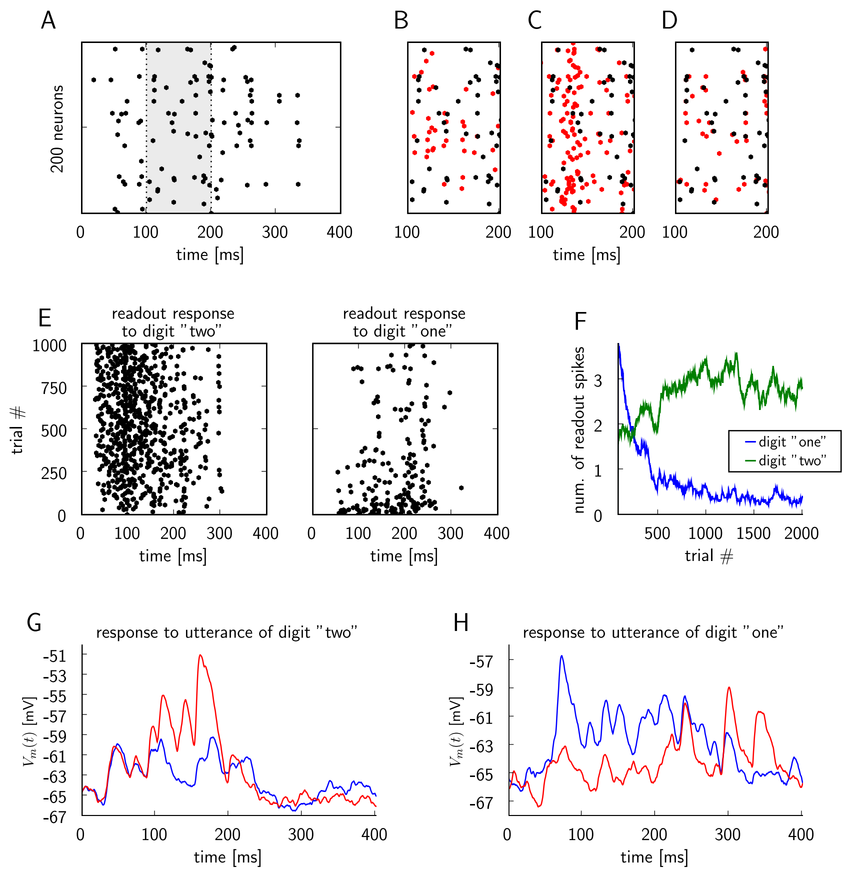

Figure S9: Variation of Fig. 10 (i.e., of computer simulation 5) for a simulation where we used current-based synapses without short-term plasticity. The post-synaptic response had an exponentially decaying form $\epsilon(s) = e^{-s/\tau_\epsilon}/\tau_\epsilon$, with $\tau_\epsilon = 5ms$. The synaptic weights of the excitatory and inhibitory synapses in the cortical microcircuit were set to $w_{exc} = 65.4$ pA and $w_{inh} = 238$ pA respectively. The maximum synaptic weight of the synapses to the readout neuron was $w_{max} = 54.3$ pA. All other parameter values were the same as in computer simulation 5.
