# Supplementary material for: A Learning Theory for Reward-Modulated Spike-Timing-Dependent Plasticity with Application to Biofeedback
Source: PLoS Comput Biol. 2008 Oct 10;4(10):e1000180. doi: 10.1371/journal.pcbi.1000180 (PMC2543108; doi:10.1371/journal.pcbi.1000180)
Supplement: Figure S11 — Spike encodings of 10 utterances of digit “two” by one speaker with the Lyon cochlea model [43], which were used as circuit inputs for computer simulation 5. (0.05 MB PDF) [file pcbi.1000180.s011.pdf]

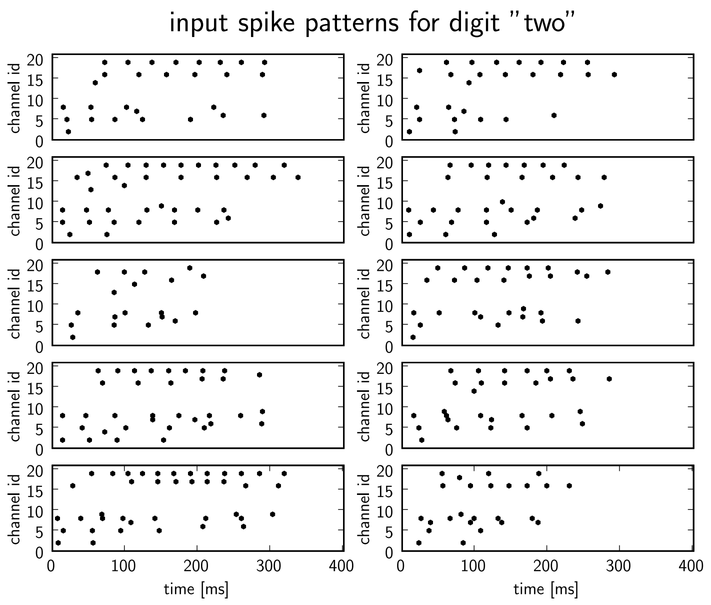

Figure S11: Spike encodings of 10 utterances of digit "two" by one speaker with the Lyon cochlea model [44], which were used as circuit inputs for computer simulation 5.
